# Walking on a User Similarity Network towards Personalized Recommendations

**DOI:** 10.1371/journal.pone.0114662

**Published:** 2014-12-09

**Authors:** Mingxin Gan

**Affiliations:** Department of Management Science and Engineering, Dongling School of Economics and Management, University of Science and Technology Beijing, Beijing, China; University of Cape Town, South Africa

## Abstract

Personalized recommender systems have been receiving more and more attention in addressing the serious problem of information overload accompanying the rapid evolution of the world-wide-web. Although traditional collaborative filtering approaches based on similarities between users have achieved remarkable success, it has been shown that the existence of popular objects may adversely influence the correct scoring of candidate objects, which lead to unreasonable recommendation results. Meanwhile, recent advances have demonstrated that approaches based on diffusion and random walk processes exhibit superior performance over collaborative filtering methods in both the recommendation accuracy and diversity. Building on these results, we adopt three strategies (power-law adjustment, nearest neighbor, and threshold filtration) to adjust a user similarity network from user similarity scores calculated on historical data, and then propose a random walk with restart model on the constructed network to achieve personalized recommendations. We perform cross-validation experiments on two real data sets (MovieLens and Netflix) and compare the performance of our method against the existing state-of-the-art methods. Results show that our method outperforms existing methods in not only recommendation accuracy and diversity, but also retrieval performance.

## Introduction

Although the rapid growth of the word-wide-web has been exposing an enormous increasing amount of commodities and information to people, information overload accompanying such resources has been recently recognized as a great challenge in both business areas and academic fields [1]. To alleviate this problem, internet search engines have been widely utilized as a fundamental technique to help people screening useful information out of a vast amount of resources. Nevertheless, a search engine, which is usually designed according to keyword-based queries of users, typically overlooks user-related historical data that in general provide valuable information about preferences of users [2]. Besides, the keyword-based design can only provide passive filtration of overloading information and lack the capability of screening useful resources in an active way [3]. To overcome these limitations, various recommender systems have been proposed to offer personalized nomination of candidate resources by assisting individuals to efficiently filtering out overload information and positively identifying their potential interest [4], which have shown great successes in a variety of applications such as the online recommendation of books [4], CDs [5], movies [6], [7], news [8], and many other resources [9].

A recommender system is usually designed based on either the collaborative filtering strategy or the content-based scheme [9]–[11]. More specifically, a user-based collaborative filtering approach uses historical data to calculate similarities between users, relies on such information to calculate discriminant scores for candidate objects, and then ranks candidates according to their scores [12], [13]. An item-based design is formally equivalent to a user-based one by simply interchanging the roles of user and objects [10]. In contrast, a content-based method characterizes similarities between objects according to their properties and then recommends to a target user new objects that are similar to those already preferred by the user [14]. In order to promote respective advantages of these two categories of approaches, hybrid approaches has also been proposed [15].

Regarding to the collaborative filtering based recommender systems, one of the most important factors determining the performance is the quantification of similarities between users, which are typically taken as a transformation of the matched relationships between object characteristics and user preferences in a recommendation process [16]. Specifically, in most existing methods, including the widely used cosine vector similarity, Jaccard index, Pearson’s correlation coefficient and recently proposed methods based on the random walk process, such a transformation is taken by representing users as vectors of objects according to the historical data of user preferences and then characterizing the similarity between two users as the similarity of corresponding vectors, which however may not cover the real relationships between users. Nevertheless, recent studies [17] have suggested that such a transformation scheme, though having been widely used by researchers to represent degrees of similarity between users, may not recover true relationships between users, because the existence of popular objects that may adversely influence the characterization of user similarities. Furthermore, it has also been demonstrated that dominant relations between users are in general effective in a recommendation [18], [19]. With this consideration, we have demonstrated that the recommendation performance of the user-based collaborative filtering approach will be greatly enhanced by either adjusting user similarities with a non-linear function or filtering out weak similarities by a nearest neighbor strategy [17].

On the other hand, recent studies have shown that recommendation approaches based on diffusion and random walk processes exhibit superior performance over classical collaborative filtering methods in not only accuracy but also diversity of recommendations [20], [21]. For instance, it has been shown that similarity measures based on the diffusion process on a user network are more accurate than the frequently-used cosine similarity measure [22], [23]. It has also been shown that the simulation of the heat-spreading process on the user-object bipartite graph can greatly improve the diversity of recommendation results [24]. Moreover, diffusion and random walk processes have also been used to adjust similarity measures [25] or construct new similarity measures [26]–[29]. However, most of these methods do not consider the influence of the underlying network structure to the effectiveness of diffusion or random walk processes.

**Figure 1:**
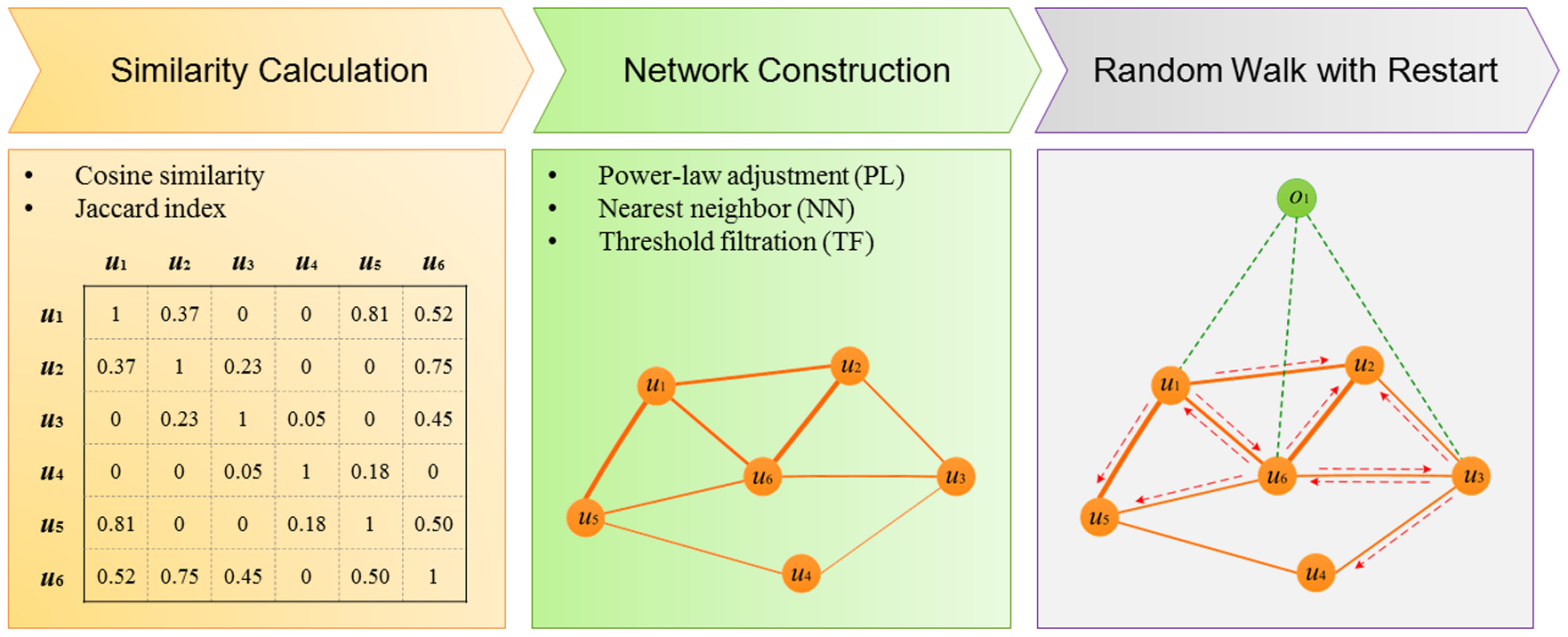
Overview of the proposed approach. We first calculate pairwise similarities between users via cosine similarity measure or Jaccard index. Then, we construct a user similarity network using one of the three strategies: power-law adjustment, nearest neighbor construction and threshold filtration. Finally, we adopt a random walk with restart model on the constructed network to facilitate the personalized recommendation of candidate objects.

Based on the above understandings and motivated by the fact that the existence of popular objects may adversely influence correct recommendations (see S1 Text and S1 Figure for detailed explanations and toy examples), we propose in this paper a random walk with restart model on a constructed user similarity network towards personalized recommendations. Specifically, as shown in Fig. 1, we first calculate user similarities based on historical data to obtain a matrix of user similarity scores. Then, we construct a user similarity network based this matrix, for the purpose of filtering out weak similarities between users. In this step, we propose three network construction strategies: power-law adjustment, nearest neighbor construction, and threshold filtration. Finally, we apply a random walk with restart model on the constructed user similarity network to calculate discriminant scores for candidate objects and further rank the objects to obtain their ranking scores. We validate our approach via comprehensive large-scale cross-validation experiments across two widely used data sets. Results show that our method remarkably outperforms existing state-of-the-art methods in not only the recommendation accuracy and diversity, but also retrieval performance. We further show the influence of parameters on the performance of our method and perform a statistical analysis to explain the reason that our method achieves high performance.

## Methods

### Construction of user similarity networks

The purpose of constructing a user similarity network instead of using all calculated similarities is to remove negative influences of some links and reveal dominant relationships. In the network construction process, we first calculate similarities between users via cosine vector similarity method. Then, we construct a user network via one of three different construction strategies, separately named *power-law adjustment*, *nearest neighbor construction* and *threshold filtration*.

Formally, given preferences of *u* users on *o* objects, represented as a matrix **X** = (*x_ij_*)*_o_*_×*u*_, where *x_ij_* = 1 if *i* is preferred by *j* and *x_ij_* = 0 otherwise, we calculate the pairwise similarity score between two users *i* and *j* using the cosine similarity, as
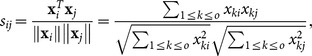
where 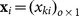 and 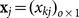 are the vectors corresponding to the *i*-th and the *j*-th users, and *s_ij_* the similarity between them. The user similarity between all users is then obtained as a matrix **S** = (*s_ij_*)*_u_*_×*u*_.

Alternatively, we compute user similarity as the Jaccard index. By treating each user as a set that containing objects preferred by the user (i.e., the set corresponds to the *i*-th user is 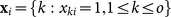), this method calculates the similarity score between two users as the number of elements in the intersection of the two sets corresponding to the two users divided by the number of elements in the union of the two sets, as
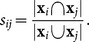



Although this matrix itself has been widely used in existing user-based collaborative filtering approaches, the existence of unreliable links may introduce much noise that typically corresponds to small user similarity scores and may adversely influence the correct calculation of discriminant scores for candidate objects. To address such a problem, we propose to filter out unreliable small user similarity scores according to the following three strategies.

#### Power-law adjustment

We apply a power-law function 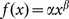 to similarity scores, yielding an adjusted user similarity profile, 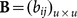, with
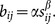



Obviously, the scaling factor *α* will be cancelled in the calculation of discriminant scores. Therefore, the only parameter is the exponent parameter *ß*. Treating users as vertices and user relationships with non-zero similarities as edges, we obtain a *ß*-power-law adjustment network.

#### Nearest neighbor construction

We remove for each user a fraction (1-*λ*) of the weakest relationships between the user and other users, obtaining a *λ*-nearest neighbor network. In detail, given a user indexed by *j*, we sort the *j*-th column of the user similarity matrix in non-ascending order to obtain ranks of the other users 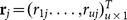. Then, we introduce a fraction *λ* and set similarity scores for users ranked lower than 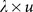 to zero. Applying the above filtering procedure to all users, we obtain a weight matrix **L** = (*l_ij_*)*_u_*_×*u*_ as
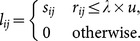



Obviously, this weight matrix corresponds to a *λ*-nearest neighbour network, in which nodes are users, and a directed edge 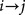 points from user *i* to user *j* if and only if *j* is among the 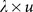 nearest neighbours of *i*.

#### Threshold filtration

We define a threshold *δ* and assign zeros to elements that are smaller than this cut-off value, obtaining a weight matrix denoted as **D** = (*d_ij_*)*_u_*_×*u*_, where
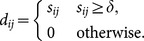



This weight matrix then corresponds to a *δ*-filtration network, in which nodes are users, and edges are non-zero relationships between users.

### Personalized recommendation by random walk with restart

We adopt a random walk with restart model on the constructed user similarity network to facilitate the recommendation of candidate objects. The basic idea of our method is to simulate the process that a random walker wanders in the user similarity network. Given a query user and a query object, the walker starts the journey at random from one of the users that have selected the query object in history. Then, in each step, the walker may either move at random to a neighboring user or start on a new journey with a certain probability. Finally, the probability that the walker stays at the query user is used as the score that reflects the preference of the query user to the query object.

Formally, given the weight matrix 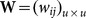 corresponding to an adjusted user similarity network (i.e., **W** = **B**, **L** or **D**), we calculate the transition matrix 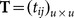 by performing a column-wise normalization to **W**, as
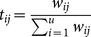



Hence, the *i*-th column in matrix **T** represents the probabilities that the random walker moves from the *i*-th user to other users. When starting a new journey, the random walker starts at random from one of the users that have selected the query object in history. We represent the initial configuration using a vector 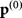, which is derive from the historical data 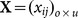 as
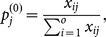
with the assumption that the query object is indexed by *i*. Then, let 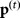 be the vector composed of probabilities that the random walker stays in all users at step *t*, we have the iteration formula



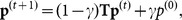
where 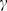 is the restart probability.

After a number of steps, the probabilities will converge to the steady state. This is obtained by performing the iteration until the difference between 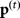 and 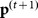 is sufficiently small (e.g., the *L*_1_ norm of 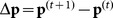 is smaller than a pre-defined small positive number *ε*). The steady-state probability 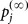 then gives a measure of the preference of the user indexed by *j* to the query object. Finally, by repeating this random walk procedure for every object, we are able to rank the objects according to the user’s preferences.

It has been shown that such a random walk model is not sensitive to parameters involved, though a relative larger restart probability benefits the performance [30]. Hence, we select default parameters as 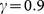 and *ε
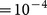
*. Moreover, for a clear presentation, we denote the random walk method on the network adjusted using power-law adjustment, nearest neighbor, and threshold filtration strategies as RWPL, RWNN and RWTF, respectively.

### Methods for comparison

We compare the proposed approach with three categories of methods. First, we implement a typical user-based collaborative filtering method named USim that weights preferences of users according to their similarities with the given user and mix the preferences to obtain discriminant scores for candidate objects [31]. Formally, given the similarity matrix **S** = (*s_ij_*)*_u_*_×*u*_, the discriminant score of a query object *s* for a query user *t* is then calculated as 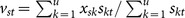. We also extend the USim method with our network construction strategies, resulting in three methods called USPL (USim with power-law adjustment, replacing **S** with **B**), USNN (USim with nearest neighbor construction, replacing **S** with **L**) and USTF (USim with threshold filtration, replacing **S** with **D**). Second, we implement two typical matrix factorization methods, non-negative matrix factorization (NMF) [32], [33] and singular value decomposition (SVD) [33], [34]. Third, we implement a probabilistic spreading method (ProbS), which works by simulating the process of reallocating resources between objects and users [35]. Formally, ProbS assigns initial resources to objects for a query user *t* as 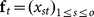 and then redistribute the resources by according to the formula 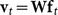 with 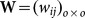 is derived as
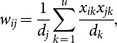
where 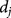 and 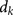 are degrees of the object *j* and user *k*, respectively [36]. The score 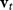 is then used to rank candidate objects.

### Validation methods and evaluation criteria

We perform 10-fold cross-validation experiments to validate the proposed approach. For this purpose, we partition known links between users and objects at random into 10 subsets of almost equal size. In each validation run, we use 9 subsets as training data to generate a user similarity matrix and use the remaining one as test data to assess the effectiveness of our method. For a certain user, we collect a set of test objects as those that connect to the user in the test data, and a set of control objects as those that neither link to the user in the training data nor in the test data. Then, we calculate discriminant scores for both the test and the control objects, and we rank each test object against all control objects in non-ascending order according to their discriminant scores. Repeating the above ranking procedure for all users, we obtain a set of ranking lists and further calculate two criteria for measuring accuracy, two criteria for evaluating retrieval performance and two criteria for assessing recommendation diversity, as defined below.

#### Accuracy measures

Given a test object and a number of control objects, we sort the test object in non-ascending order according to their discriminant scores. In the situation that multiple objects have equal discriminant scores, we break the tie by putting these objects in random order. We further divide the rank by the total number of test and control objects to obtain the relative rank. Then, we average relative ranks for all objects in the test set to obtain the criterion named *mean relative rank*. Obviously, this criterion measures the accuracy of a method in recommending user preferred objects, and a method with high accuracy tends to have a lower mean relative rank.

Given a threshold *L* (defaulting to 20 in this paper, the same in the calculation of other criteria), we claim a test case as true-positive (TP) if it is ranked among top *L*, and we claim a control case as false-positive (FP) if it is ranked among top *L*. Then, we calculate a criteria denoted by *precision* @ *L* as TP/(TP+FP). Obviously, a method with high accuracy tends to have a lower mean relative rank and a higher precision @ *L*.

#### Retrieval measures

Given a pre-defined threshold *L*, we claim a test object as successfully recommended if the object has been ranked among top *L* in the ranking list. For a user *u_j_* who has collected a number of *D_j_* objects in the test data, we count the number of successful recommendations among these objects as *R_j_* and calculate the fraction of successfully recommended objects to obtain the recall for the user, as 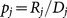. Finally, averaging over recalls for all users who have collected at least one object in the test data, we obtain the recall under the threshold *L*, denoted by *RE*(*L*). To take into account intrinsic properties of the data, we further compare a recommender method with the random guess approach. By random guess, the probability that a test object ranks among top *L* for user *u_j_* is 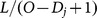, and the expected number of successful recommendations is 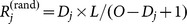, resulting in a recall of 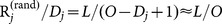 and an average recall of 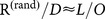, since in general total number of objects 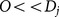. We then define recall enhancement as the fold enhancement of the recall over the random guess approach, as
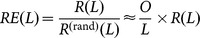



In this paper, we use *L* = 20 in the calculation of this criterion. It is also obvious that a method of higher recommendation accuracy will have a larger recall enhancement.

We also adopt another commonly used retrieval criterion named *hit-rate @ L.* Given a threshold *L*, we claim a test case as successfully hit if it is ranked among top *L*. Calculating the fraction of hit cases for every user and average over all users, we obtain the criteria value in hit-rate @ *L* [35].

#### Diversity measures

The first criterion for evaluating recommendation diversity is called *mean personalization* (MP). Given discriminant scores calculated for a list of objects, we sort the objects in descending order according to their scores and obtain a subset of objects, 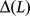, that are ranked among top *L* in the ranking list. For two users *j* and *k*, we count the number of objects shared by their corresponding top-ranking sets, 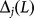 and 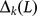, and further normalize this number by the threshold value *L* to obtain the degree of overlap between the two ranking lists. Finally, we define the mean personalization as one minus the average degree of overlap between every two users, as
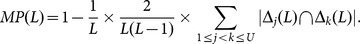



The second criterion for evaluating the diversity is called *mean novelty* (MN). For each object, we calculate the fraction of users that have collected the object, and obtain the information content of the object as the negative logarithm of the fraction. Then, given the top-ranking subset of objects for a *j*-th user as 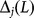, we average over the information content of the objects in the set to obtain the novelty of recommendation for the user. Finally, we define mean novelty as the average novelty over all users, as
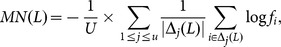
where *f_i_* is the fraction of users that have collected the *i*-th object.

## Results

### Data sources

We used two large-scale data sources to validate the proposed approach. The first data set was called MovieLens, obtained from the GroupLens lab (http://www.grouplens.org). The original data set included more than 10 million ratings given by 69,878 users for 10,677 movies. Each rating had 10 values, ranging from 0.5 (worst) to 5.0 (best) with step 0.5. We first down-sampled at random 5,000 users from the original data and retained 5,977 movies rated by at least 5 of such users. Then, we followed the literature [37] to convert the ratings to binary links by assigning 1 as “relevant” to ratings no less than 3.0 and 0 as “not-relevant” to all other cases. Finally, we obtained a data set that included 581,731 links between 5,000 users and 5,977 movies. We referred to this data set as “MovieLens” in the rest of this paper.

The second data set, called Netflix, was obtained from the Netflix Prize (http://www.netflixprize.com). This data set contained about 100 million ratings given by 480,189 users for 17,770 movies. Each rating had 5 possible values, ranging from 1 (worst) to 5 (best) with step 1. We performed a similar sampling process by down-sampling at random 5,000 users and retaining 4,555 movies rated by at least 10 of such users. Treating ratings below 3.0 as “not-relevant” and those above 3.0 as “relevant”, we obtained a data set that includes 294,387 links between the sampled users and movies. We referred to this data set as “Netflix” in the rest of this paper.

### Enrichment of test objects among top of ranking lists

**Figure 2:**
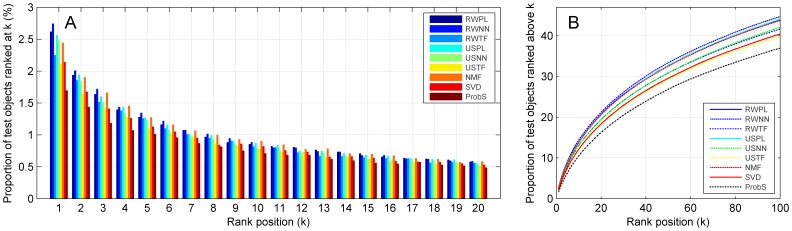
Enrichment of test objects among top rank positions. (A) Proportions of test objects ranked at top positions. (B) Cumulative distributions of top ranking objects. Results are obtained by 10-fold cross-validation experiments on MovieLens (5,000 users and 5,977 objects) with cosine similarity measure. Restart probabilities for random walk approaches are set to 0.9.

With the understanding that an effective recommender system should rank user preferred objects among top positions of a ranking list, we focused on MovieLens and the cosine similarity measure to perform the validation experiment as detailed in the section of methods, and we investigated the proportion of test objects that occupied exactly the *k*-th position of the final ranking lists 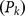. As shown in Fig. 2(A), the random walk approach, when used with a properly constructed user similarity network, exhibits superior capability in enriching test objects among top positions. For example, when used with the power-law adjusted network (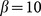, 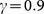), RWPL ranks 2.62% test objects at the top, 1.90% at second, 1.67% at third, and so on. When used with nearest neighbor network (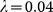, 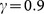), RWNN ranks 2.80% test objects at the top, 2.05% at second, 1.72% at third, and so on. When used with the threshold filtration network (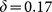, 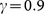), RWTF ranks 2.26% test objects at the top, 1.90% at second, 1.51% at third, and so on. Since on average a random guess procedure can only rank about 0.017% (1/(5977−581731/5000)×100%) objects at the top, the effectiveness of the proposed approach is strongly supported.

We further calculated the cumulative distribution of top ranking test objects by calculating the proportion of test objects ranked higher than or equal to a position (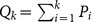). As shown in Fig. 2(B), the curves of RWPL and RWNN stay above the other methods, suggesting the superior performance of these two approaches. We also notice that methods based on the random walk model in general outperform those based on the collaborative filtering methods, because the cumulative distribution curves of RWPL, RWNN and RWTF stay above those of USPL, USNN and USTF, respectively.

We also saw that the network-based approaches exhibit much higher performance than methods without using networks. For example, NMF can rank 2.45% test objects at the top, 1.90% at second, 1.62% at third. SVD ranks 2.06% test objects at the top, 1.67% at second, 1.45% at third. ProbS can rank 1.68% test objects at the top, 1.50% at second, 1.17% at third. All these results are obviously worse that both RWPL and RWNN.

We finally adopted Binomial exact tests to compare performance of different methods. For a certain user, we claim method A having higher performance than B if ranking positions of more than half test objects generated by A are ahead of those provided by B. Then, we count the number of users for whom method A outperforms B and test whether the relative frequency of such users is greater than 0.5 according to a one-sided Binomial exact test. Results, as summarized in Table 1, suggest that the random walk approaches in general perform significantly higher than their collaborative filtering counterparts. That is, RWPL outperforms USPL; RWNN outperforms USNN; RWTF outperforms USTF. Furthermore, all methods based on random walk (RWPL, RWNN, RWTF) performs significantly higher than NMF, SVD and ProbS. These observations suggest that the random walk methodology can indeed improve recommendation performance. Moreover, all methods based on network construction (RWPL, RWNN, RWTF, USPL, USNN, USTF) perform significantly higher than NMF, SVD and ProbS, suggesting the effectiveness of the network construction strategies. Among the three network construction strategies, power-law adjustment owns the highest performance, followed by nearest neighbor construction and threshold filtration. As for the methods without network construction strategies, NMF owns the highest performance, followed by SVD and then ProbS. For individual methods, RWPL exhibits the highest performance by outperforming USPL at the marginal significance level of 0.1 and all other methods at the significance level of 10^−8^, all after the Bonferroni correction for multiple comparisons. USPL, as the method of the second highest performance, outperforms all other methods except for RWPL at the significant level of 10^−8^ after the Bonferroni correction.

**Table 1:** Comparison of different methods.

Method	RWPL	RWNN	RWTF	USPL	USNN	USTF	NMF	SVD	ProbS
RWPL		***	***	*	***	***	***	***	***
RWNN	+		***	+	***	***	***	***	***
RWTF	+	+		+	+	***	***	***	***
USPL	+	***	***		***	***	***	***	***
USNN	+	+	***	+		***	***	***	***
USTF	+	+	+	+	+		*	***	***
NMF	+	+	+	+	+	+		***	***
SVD	+	+	+	+	+	+	+		+
ProbS	+	+	+	+	+	+	+	*	

Results are obtained by 10-fold cross-validation experiments on MovieLens (5,000 users and 5,977 objects) with cosine similarity measure. * and *** denotes the method in the corresponding row is better than that in the corresponding column at the statistical significance level of 10^−1^ and 10^−8^ after the Bonferroni correction, respectively.+denotes the null-hypothesis cannot be rejected at the significance level of 10^−1^ after the Bonferroni correction. Restart probabilities for random walk approaches are set to 0.9. Parameters are set to *ß* = 10 for RWPL, *λ* = 0.04 for RWNN, *δ* = 0.17 for RWTF, *ß* = 10 for USPL, *λ* = 0.03 for USNN and *δ* = 0.20 for USTF to obtain the highest performance.

### Improvement of the recommendation performance

We then evaluated each method using the aforementioned six criteria and summarized the results in Table 2. From the table, we observe that the random walk approach with a properly constructed user similarity network outperforms the other methods in not only recommendation accuracy and diversity but also retrieval performance. For example, when used with power-law adjusted network (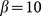, 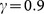), RWPL achieves a mean relative rank of 7.25%, a precision at the default *L* value of 20 of 14.65%, a recall enhancement of 99.03, a hit-rate at *L* = 20 of 70.78%, a mean personalization of 87.85%, and a mean novelty of 2.58. When used with nearest neighbor network (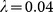, 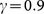), RWNN achieves a mean relative rank of 7.25%, a precision at *L* = 20 of 15.21%, a recall enhancement of 102.56, a hit-rate at *L* = 20 of 72.50%, a mean personalization of 83.01% and a mean novelty of 2.32. When used with threshold filtration network (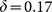, 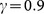), RWTF achieves a mean relative rank of 8.66%, a precision at *L* = 20 of 13.73%, a recall enhancement of 94.38, a hit-rate at *L* = 20 of 69.82%, a mean personalization of 79.38%, and a mean novelty of 2.36.

**Table 2:** Performance of different methods.

Method	*MRR* (%)	*PR@20* (%)	*RE*	*HR@20* (%)	*MP* (%)	*MN*
RWPL (*ß* = 10)	**7.25** **(0.07)**	14.65 (0.05)	99.03 (0.58)	70.78 (0.43)	**87.85 (0.21)**	**2.58 (0.04)**
RWNN (*λ* = 0.04)	**7.25 (0.06)**	**15.21 (0.07)**	**102.56 (0.47)**	**72.50 (0.42)**	83.01 (0.23)	2.32 (0.04)
RWTF (*δ* = 0.17)	8.66 (0.08)	13.73 (0.08)	94.39 (0.67)	69.82 (0.38)	79.38 (0.22)	2.36 (0.05)
USPL (*ß* = 10)	7.27 (0.05)	14.63 (0.06)	98.82 (0.71)	70.80 (0.47)	87.81 (0.21)	2.57 (0.06)
USNN (*λ* = 0.03)	7.74 (0.07)	14.04 (0.05)	94.30 (0.75)	69.42 (0.49)	74.82 (0.24)	2.06 (0.10)
USTF (*δ* = 0.20)	9.61 (0.06)	13.03 (0.04)	91.31 (0.78)	68.45 (0.43)	75.78 (0.21)	2.27 (0.09)
NMF	7.70 (0.08)	14.53 (0.06)	93.78 (0.67)	68.39 (0.48)	80.57 (0.20)	2.16 (0.08)
SVD	8.69 (0.09)	12.85 (0.05)	83.06 (0.53)	63.77 (0.37)	74.16 (0.17)	2.03 (0.05)
ProbS	9.03 (0.07)	11.29 (0.07)	78.51 (0.67)	61.09 (0.27)	46.05 (0.25)	1.73 (0.13)

Results are mean (standard derivation) obtained by 10-fold cross-validation experiments on MovieLens (5,000 users and 5,977 objects) with cosine similarity measure. Restart probabilities for random walk approaches are set to 0.9. *MRR* represents mean relative rank, *PR@20* represents precision at the default *L* value of 20, *RE* represents recall enhancement, *HR@20* represents hit-rate at *L* = 20, *MP* represents mean personalization, *MN* represents mean novelty.

We compared the recommendation performance of these random walk approaches with their collaborative filtering counterparts (USPL, USNN and USTF) and clearly saw the superior performance of the former. When used with nearest neighbor strategy, the best performance of RWNN (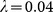, 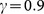) outperforms that of USNN (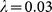) by 0.51 (6.60%) in terms of mean relative rank, 1.20% (8.57%) in precision at *L* = 20, 8.26 (8.76%) in recall enhancement, 3.10% (4.47%) in hit-rate at *L* = 20, 8.19% (10.95%) in mean personalization and 0.26 (12.62%) in mean novelty. One-sided Wilcoxon rank sum tests also support the superiority of RWNN over USNN (the *p*-value based on any of the criteria is less than 10^−8^).

When used with threshold filtering strategy, the best performance of RWTF (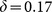, 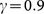) outperforms that of USTF (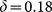) by 0.80% (8.46%) in terms of mean relative rank, 0.98% (7.70%) in precision at *L* = 20, 4.02 (4.46%) in recall enhancement, 1.85% (2.73%) in hit-rate at *L* = 20, 6.90% (9.56%) in mean personalization and 0.19 (8.60%) in mean novelty. One-sided Wilcoxon rank sum tests also support the superiority of RWTF (the *p*-value based on any of the criteria is less than 10^−8^). We also notice that the network-based approaches demonstrate much higher performance than methods without using networks. For example, the existing state-of-the-art matrix factorization method NMF achieves a mean relative rank of 7.68%, a precision at *L* = 20 of 14.43%, a recall enhancement of 93.25, a hit-rate at *L* = 20 of 68.06%, a mean personalization of 80.23%, and a mean novelty of 2.16. By comparison, the improvement of RWNN over NMF is as high as 0.45% (5.95%) in mean relative rank, 0.74% (5.17%) in precision at *L* = 20, 8.50 (9.11%) in recall enhancement, 4.28% (6.28%) in hit-rate at *L* = 20, 2.78% (3.47%) in mean personalization, and 0.17 (7.73%) in mean novelty. One-sided Wilcoxon rank sum tests also support the superiority of RWNN over NMF (the *p*-value based on any of the criteria is less than 10^−8^). The existing state-of-the-art method ProbS achieves a mean relative rank of 9.03%, a precision at *L* = 20 of 11.20%, a recall enhancement of 77.97, a hit-rate at *L* = 20 of 60.67%, a mean personalization of 45.69%, and a mean novelty of 1.73. By comparison, the improvement of RWNN over ProbS is as high as 1.81% (19.99%) in mean relative rank, 3.98% (35.59%) in precision at *L* = 20, 23.77 (30.48%) in recall enhancement, 11.68% (19.25%) in hit-rate at *L* = 20, 37.33% (81.69%) in mean personalization, and 0.60 (34.57%) in mean novelty. One-sided Wilcoxon rank sum tests also support the superiority of RWNN (the *p*-value based on any of the criteria is less than 10^−8^).

### Influence of network construction strategies and related parameters

**Figure 3:**
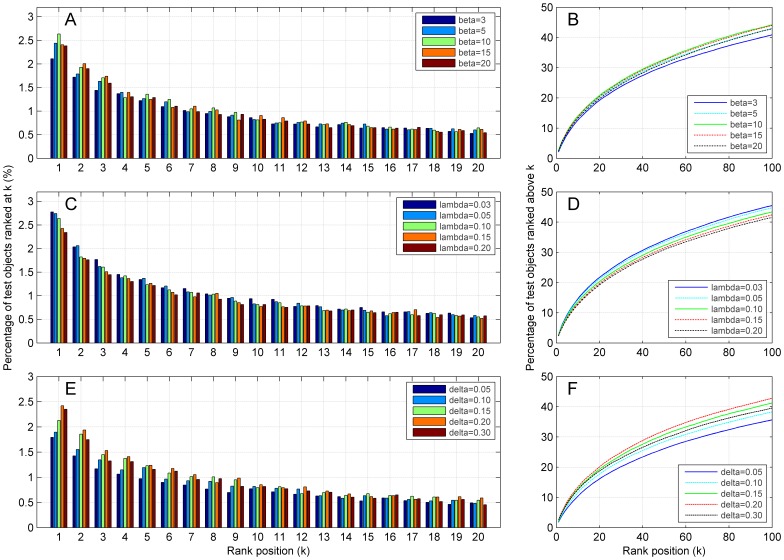
Influence of network construction strategies and related parameters on proportions of top ranking test objects. (A) Proportions of test objects ranked at top positions. (B) Cumulative distributions of top ranking objects. Results are obtained by 10-fold cross-validation experiments on MovieLens (5,000 users and 5,977 objects) with cosine similarity measure. Restart probabilities for random walk approaches are set to 0.9.

We assessed how different network construction strategies and related parameters influence the recommendation performance of the random walk approach, also based on the MovieLens dataset. For this purpose, we fixed the restart probability 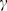 to 0.9, varied the exponent coefficient (*ß*) in power-law adjustment strategy from 1 to 20 and summarized the proportion of test objects enriched among top positions in Fig. 3 (A and B). We observe from the figure that a *ß* value around 10 maximizes the capability of the proposed method in enriching test objects among top positions, and either smaller or larger *ß* values impair the performance. Similarly, by varying the proportion of users (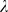) in nearest neighbor strategy from 0 to 0.2, we observe that the optimal 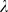 value is around 0.03 (Fig. 3, C and D) and either smaller or larger 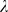 values impair the performance. By varying the cutoff value (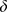) in threshold filtration strategy from 0 to 0.3, we observe the same phenomenon that either small or large 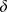 values impair the capability of the random walk method in enriching top ranking test objects, and a 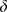 value around 0.20 maximizes the performance (Fig. 3, E and F).

**Figure 4:**
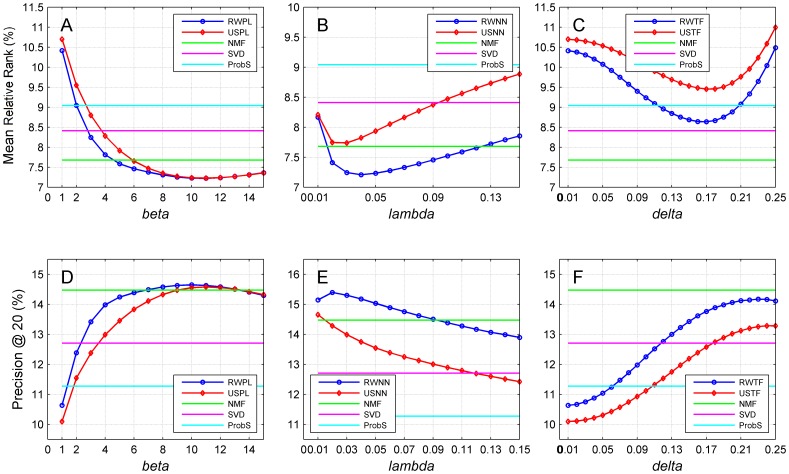
Performance of the proposed methods with related parameters of three network construction strategies on recommendation accuracy criteria. (A–C) Mean relative rank. (D–F) Precision at *L* = 20. Results are obtained by 10-fold cross-validation experiments on MovieLens (5,000 users and 5,977 objects) with cosine similarity measure. Restart probabilities for random walk approaches are set to 0.9. The lower the mean relative rank, the better the performance of recommendation accuracy. The higher the precision at *L* = 20, the better the recommendation accuracy.

We then studied how the recommendation accuracy is affected by the network construction strategies and summarized the results in Fig. 4. We observe clearly from this figure (A and D) that both the mean relative rank and the recall enhancement for the random walk approach with power-law adjusted network (RWPL) improve at a sharp ratio with the increase of *ß* and then drop gently with the further increase of *ß*. For example, as *ß* varies from 1 to 20, the mean relative rank improves drastically when 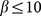, achieves the optimal value of 7.25% at 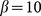, and then decreases gradually. The collaborative filtering method with power-law adjusted network (USPL) exhibits a similar pattern and achieves the optimal mean relative rank of 7.25% at 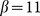. NMF, SVD and ProbS keep constant mean relative ranks of 7.70%, 8.69% and 9.03% separately, regardless the varying of *ß*. Similarly, Fig. 4 (B and E) reflects how the two accuracy criteria change with different 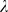 values in nearest neighbor strategy, and Fig. 4 (C and F) reflects how recommendation accuracy changes with various 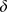 values in threshold filtration strategy. From these subplots, we observe similar patterns as those exhibiting in the analysis of power-law adjusted network (Fig. 4, A and D).

**Figure 5:**
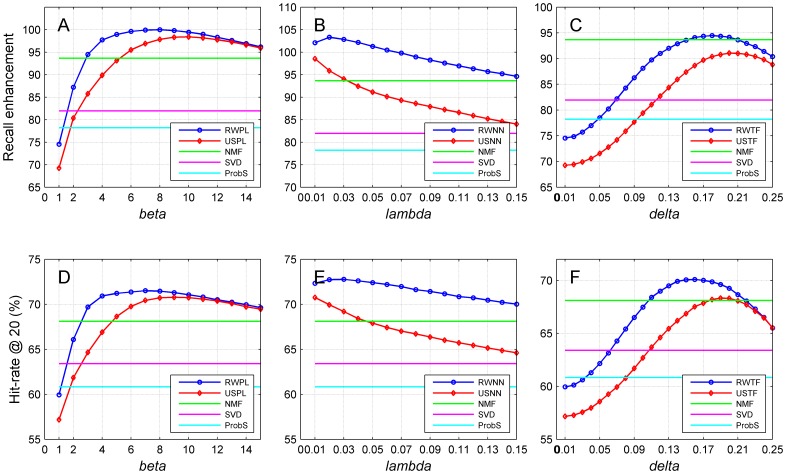
Performance of the proposed methods with related parameters of three network construction strategies on recommendation retrieval criteria. (A–C) Recall enhancement. (D–F) Hit-rate at *L* = 20. Results are obtained by 10-fold cross-validation experiments on MovieLens (5,000 users and 5,977 objects) with the cosine similarity measure. Restart probabilities for random walk approaches are set to 0.9. The higher the recall enhancement, the better the recommendation retrieval performance. The higher the hit-rate at *L* = 20, the better the retrieval performance.

We further studied how the retrieval performance is affected by the network construction strategies and summarized the results in Figure 5. For the random walk approach with power-law adjusted network (RWPL, subplots A and D), as *ß* increases, the retrieval related metrics (recall enhancement and hit-rate at *L* = 20) first increase rapidly and then gradually decrease. For example, the recall enhancement achieves the optimal value of 100.03 as 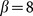. The collaborative filtering method with power-law adjusted network (USPL) exhibits a similar pattern and achieves the optimal recall enhancement of 98.39 as 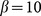. NMF, SVD and ProbS keep constant recall enhancements of 93.66, 81.93 and 78.21 separately, regardless the varying of *ß*. Similarly, Fig. 5 (B and E) reflects how the two retrieval criteria change with different 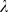 values in nearest neighbor strategy, and Fig. 5 (C and F) reflects how recommendation retrieval performance changes with various 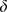 values in threshold filtration strategy. From these subplots, we observe similar patterns as those exhibiting in the analysis of power-law adjusted network (Fig. 5, A and D).

**Figure 6:**
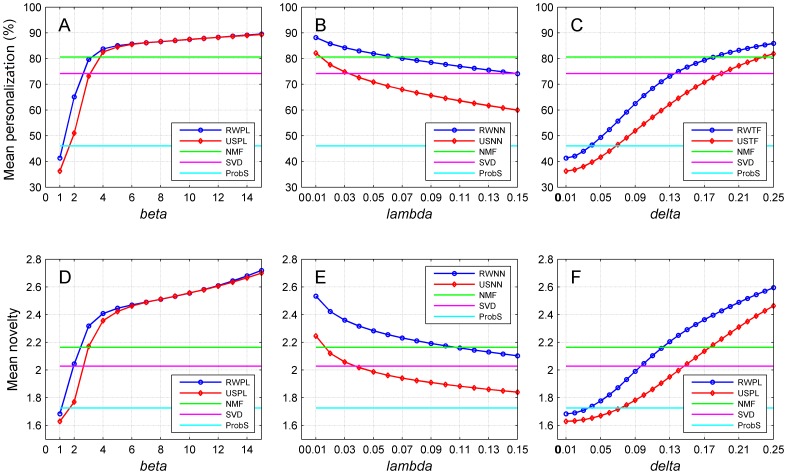
Performance of the proposed methods with related parameters of three network construction strategies on recommendation diversity criteria. (A–C) Mean personalization. (D–F) Mean novelty. Results are obtained by 10-fold cross-validation experiments on MovieLens (5,000 users and 5,977 objects) with cosine similarity measure. Restart probabilities for random walk approaches are set to 0.9. The higher the mean personalization, the better the recommendation diversity performance. The higher the mean novelty, the better the diversity performance.

We further studied how the recommendation diversity is affected by the network construction strategies and summarized the results in Fig. 6. For the random walk approach with power-law adjusted network (RWPL, subplots A and D), as *ß* increases, the diversity related metrics (mean personalization and mean novelty) first increase rapidly and then gradually stabilize. Finally, the mean personalization reaches 90.51% and the mean novelty reaches 2.97 at *ß = *20. For the collaborative filtering method with power-law adjusted network (USPL, subplots A and D), we observe a similar pattern and obtain a mean personalization of 90.22% and a mean novelty of 2.94 at *ß = *20. NMF, SVD and ProbS keep constant mean personalization of 90.31%, 89.63% and 80.98% separately, and constant mean novelties of 3.51, 3.75 and 3.42 separately, regardless the varying of *ß*. We observe similar patterns for nearest neighbor strategy (Fig. 6, B and E) and threshold filtration strategy (Fig. 6, C and F).

One of the main advantages of our network-based recommendation approach is to reduce adverse influences of weak relationships between users. In power-law adjustment strategy, this goal is achieved by using the parameter *ß* to enlarge the difference between strong relationships and weak associations between users. When *ß* = 0, the resulting network degenerates to a fully connected unweighted network, and both random walk and collaborative filtering based on such a network degenerate to the Global Rank method [31]. When *ß* = 1, the network corresponds to the original user similarity matrix. When *ß* = ∞, the resulting network degenerates to a disconnected network with no edge, and both random walk and collaborative filtering based on such a network degenerate to random guess. In the middle of the spectrum, with a properly selected value of *ß*, the power of small values in the user similarity matrix tends to be zero more quickly than those of large values, resulting in an effect that is equivalent to the removal of weak associations between users.

With similar reasoning, nearest neighbor network corresponds to the original user similarity matrix when 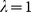 and degenerates to a disconnected one when 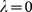. With a properly selected value of 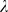, only a small fraction of strong relationships between users is kept, also equivalent to the removal of weak associations. Similarly, threshold filtration network corresponds to the original user similarity matrix when 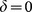 and degenerates to a disconnected one when 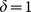. With a properly selected value of 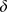, only a small fraction of strong relationships exceeding the threshold is kept, corresponding to the removal of weak associations.

### Statistical explanation of the improvement in recommendation performance

**Figure 7:**
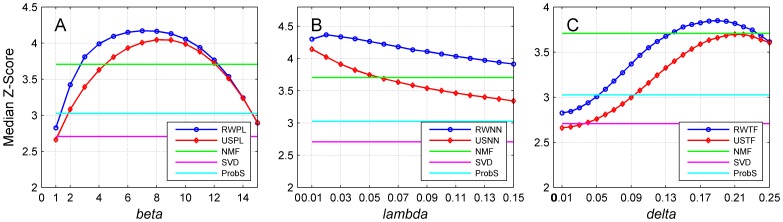
Performance of the proposed methods with related parameters of three network construction strategies on the median z-score. Results are obtained by 10-fold cross-validation experiments on MovieLens (5,000 users and 5,977 objects) with the cosine similarity measure. Restart probabilities for random walk approaches are set to 0.9.

We further conducted a statistical analysis on the reason that the accuracy metrics as illustrated in Fig. 4 exhibited an improvement and then decline pattern. For this purpose, we estimated for each user the mean and standard deviation of discriminant scores for control objects. Then, for each test object related to a user, we subtracted the mean from its discriminant score and divided the difference by the standard deviation to obtain a z-score. Finally, we identified the median of all such z-scores to obtain an index called the median z-score. By varying the associated parameters for each network construction strategy, we plotted the median z-score in Fig. 7. From subplot A of this figure, we observe that for the random walk method on power-law adjusted network (RWPL), the median z-score increases drastically as *ß* increases and then drops gradually as *ß* keeps increasing. Compared with ProbS, the improvement of the median z-score at the optimal point (*ß* = 7) is about 42%. We also observe that median z-score for the collaborative filtering approach with power-law adjusted network (USPL) also exhibits a similar increasing and then decreasing pattern. Nevertheless, when *ß* 12, the z-score value of USPL is less than that of RWPL at the same value of *ß*. We also notice from Fig. 7 (B and C) that median z-scores for the other two network construction strategies demonstrate similar patterns as that for power-law adjustment strategy.

These observations are consistent with our previous results (Fig. 4) regarding the improvement of the recommendation accuracy and can be explained as follows. In the recommendation process, the rank of a test object is determined by comparing its discriminant score with those of control objects. Hence, the larger the z-score for a test object, the higher the degree that the test object deviates from the control objects, and thus the higher the rank that the test object is likely to receive. Considering all test objects as a whole, the median z-score reflects how likely test objects receive high ranks. More precisely, a large median z-score indicates that the test objects are likely to be ranked high, and thus the recommendation accuracy is likely to be high. For example, from Fig. 7 (A), we see that the trend of the median z-score against *ß* demonstrates the increasing and then decreasing pattern. Consequently, the recommendation accuracy as illustrated in Fig. 4 (A and D) also exhibits the increasing and then decreasing pattern.

### Influence of the random walk parameters

**Figure 8:**
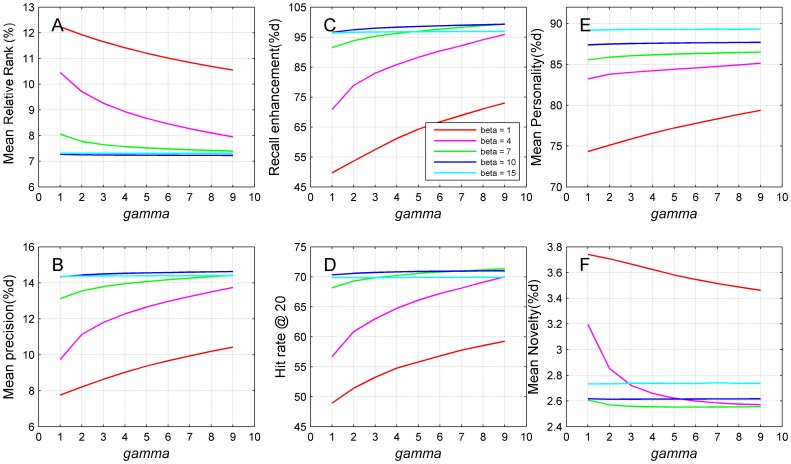
Influence of the restart probability on the recommendation performance of the random walk method with power-law adjustment network. (A) Mean relative rank. (B) Precision at *L* = 20. (C) Recall enhancement. (D) Hit-rate at *L* = 20. (E) Mean personalization. (F) Mean novelty. Results are obtained by 10-fold cross-validation experiments on MovieLens (5,000 users and 5,977 objects) with cosine similarity measure. Restart probabilities for random walk approaches are set from 0.1 to 0.9.

The behavior of the random walk with restart model is controlled by the restart parameter. Intuitively, with a large restart probability, the random walker cannot go far away from the starting point, mimicking the style of a conservative walker, while with a small restart probability, the walker is able to travel a long distance towards nodes far away from the starting point, simulating the manner of a radical walker. As an extreme case, a restart probability of 1.0 means that the walker always stay at the starting point and cannot go to any other node in the network. Consequently, all pairs of objects and users in query will be assigned zero scores, and the recommendation process is therefore equivalent to a random guess approach. We then studied the influence of the restart probability to the performance of the proposed approach by varying this parameter from 0.0 to 0.9 and summarized the results in Fig. 8 (for power-law adjusted network).

From the figure (A and B), we observe that a small restart probability typically impairs the accuracy of the recommendation, while a large restart probability in general results in high recommendation accuracy. Besides, our approach is quite robust to this parameter in a wide range of relatively large values. For example, with the restart probability 0.1, the random walk model with power-law adjusted network (RWPL, 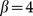) achieves a mean relative rank of 10.45%. When the restart probability increases to 0.2, the mean relative rank improves rapidly to 9.77%. When the restart probability keeps increase, the recommendation accuracy keeps improving, but the extent of improvement tends to be small. When the restart probability is greater than or equal to 0.8, differences in accuracy metrics at various parameter values become almost negligible, suggesting the robustness of our approach to the restart probability. This observation reflects a merit feature of our approach and brings convenience to the selection of this parameter. We can roughly select a relatively large restart probability in the range of 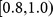 without tuning for the optimal parameter value. The same consideration holds for both nearest neighbor network and threshold filtration network. From Fig. 8 (C and D, E and F), we observe that both of the recommendation retrieval and diversity criteria exhibit similar patterns to the recommendation accuracy criteria.

### Consistency between different similarity measures and different data sets

Although the cosine similarity measure has been widely used in the calculation of user similarity scores, there also exist several other methods for the same purpose. We therefore ask the question of whether the observed improvements in recommendation accuracy, diversity and retrieval performance are consistent between different methods for calculating user similarity scores. To answer this question, we replaced cosine similarity with Jaccard index and repeated all the above experiments. Results, as detailed in S1 Text, S2, S3, S4 Figures and S1 Table, suggest that the superior performance of the random walk model is consistent between different methods for calculating user similarities.

It is also natural to ask the question of whether improvements achieved by our method are consistent between different data sets. To answer this question, we replace MovieLens with Netflix (5,000 users and 4,555 objects) and repeat all the validation experiments with the use of the cosine similarity measure (S1 Text, S5, S6, S7 Figures, and S2 Table) and the Jaccard index measure (S1 Text, S8, S9, S10 Figures and S3 Table). Results suggest that the improvement in recommendation performance on Netflix is consistent with what exhibited on MovieLens.

Finally, we ask the question of whether the above conclusions are still valid for relatively large data sets. To answer this question, we increase the number of both sampled users and objects to 10,000 on MovieLens and repeat the validation experiments. From the results detailed in the S1 Text, S4, S5 Tables, we observe similar patterns for accuracy, retrieval and diversity criteria on the large data sets, suggesting that the previous conclusions are independent of the number of users and objects sampled.

## Conclusions and Discussion

We have proposed a random walk with restart approach on constructed user similarity network towards personalized recommendation and demonstrated the superior performance of this approach over existing state-of-the-art methods via large-scale validation experiments. We have summarized the enhancement of our approach in not only the accuracy and retrieval performance, but also the diversity of recommendation results. We have also shown that the performance of the proposed method is consistent over two methods for calculating user similarities across two widely used data sets.

Our method achieves outstanding performance mainly due to the combination of two aspects. First, our network construction strategies emphasize strong relationships or remove weak relationships between users. Such relationships with weak association or irrelevant links adversely affect the correct calculation of discriminant scores for candidate objects in the ordinary collaborative filtering approach. Our methods, as demonstrated comprehensively, can effectively reduce such adverse influence with the appropriate construction of user similarity networks through three different construction strategies. Second, the random walk with restart model further utilizes user similarity networks in a more effective way than the ordinary collaborative filtering approach. As a result, our method achieves significant improvements in the accuracy, retrieval and diversity of resulting recommendations, while only adding few computational burdens. Consequently, our method is ready to be used in recommender systems that are based on the ordinary user-based framework to achieve easy yet reasonable improvements.

Certainly, the proposed method can be further investigated from the following aspects. First, although our method is aimed at improving the random walk methods for user network, it is straightforward to incorporate the principles of our method into the item-based random walk methods. This can be done by constructing an object similarity network by applying power-law adjustment strategy, nearest neighbor or threshold filtration strategies. The idea of constructing similarity networks can also be incorporated into content-based methods. Second, it is also possible to integrate a user similarity network and an object similarity network to construct a heterogeneous network and then apply the random walk model or graph algorithms [38] on such a network. Third, we have provided simulation studies and comprehensive analysis about the influence of power-law adjustment parameter (*ß*), nearest neighbors fraction (*λ*) and filtration threshold (*δ*) on the performance of the proposed methods. However, theoretical analysis about the optimal values of these parameters is left open. We will pursue this theoretical goal in our future work.

## Supporting Information

S1 Figure **Effects of power law adjustment, nearest neighbor construction and threshold filtering to user similarity scores.** A: Two objects *o*_1_ and *o*_2_ are assigned equal scores by random walk with restart probability at 0.9. B: *o*_1_ is assigned a smaller score than *o*_2_ by random walk with restart probability at 0.9. C: In contrast to (A), *o*_1_ is assigned a larger score than *o*_2_ after applying power-law adjustment (*ß* = 2) to user similarity scores. D: In contrast to (B), *o*_1_ is assigned a larger score than *o*_2_ after applying power-law adjustment (*ß* = 2) to user similarity scores. E: In contrast to (A), *o*_1_ is assigned a larger score than *o*_2_ after applying nearest neighbor construction (*λ* = 10%) to user similarity scores. F: In contrast to (B), *o*_1_ is assigned a larger score than *o*_2_ after applying nearest neighbor construction (*λ* = 10%) to user similarity scores. G: In contrast to (A), *o*_1_ is assigned a larger score than *o*_2_ after applying threshold filtering (*δ* = 0.20) to user similarity scores. H: In contrast to (B), *o*_1_ is assigned a larger score than *o*_2_ after applying threshold filtering (*δ* = 0.20) to user similarity scores.
(TIF)

S2 Figure **Performance of the proposed methods with related parameters of three network construction strategies on recommendation accuracy criteria**. (A–C) Mean relative rank. (D–F) Precision at *L* = 20. Results are obtained by 10-fold cross-validation experiments on MovieLens (5,000 users and 5,977 objects) with the Jaccard index measure. Restart probabilities for random walk approaches are set to 0.9. The lower the mean relative rank, the better the performance of recommendation accuracy. The higher the precision at *L* = 20, the better the recommendation accuracy.
(TIF)

S3 Figure **Performance of the proposed methods with related parameters of three network construction strategies on recommendation retrieval criteria**. (A–C) Recall enhancement. (D–F) Hit-rate at *L* = 20. Results are obtained by 10-fold cross-validation experiments on MovieLens (5977 objects and 5000 users) with Jaccard index. Restart probabilities for random walk approaches are set to 0.9. The higher the recall enhancement, the better the recommendation retrieval performance. The higher the hit-rate at *L* = 20, the better the retrieval performance.
(TIF)

S4 Figure **Performance of the proposed methods with related parameters of three network construction strategies on recommendation diversity criteria**. (A–C) Mean personalization. (D–F) Mean novelty. Results are obtained by 10-fold cross-validation experiments on MovieLens (5977 objects and 5000 users) with Jaccard index. Restart probabilities for random walk approaches are set to 0.9. The higher the mean personalization, the better the recommendation diversity performance. The higher the mean novelty, the better the diversity performance.
(TIF)

S5 Figure **Performance of the proposed methods with related parameters of three network construction strategies on recommendation accuracy criteria**. (A–C) Mean relative rank. (D–F) Precision at *L* = 20. Results are obtained by 10-fold cross-validation experiments on Netflix (4555 objects and 5000 users) with cosine similarity measure. Restart probabilities for random walk approaches are set to 0.9. The lower the mean relative rank, the better the performance of recommendation accuracy. The higher the precision at *L* = 20, the better the recommendation accuracy.
(TIF)

S6 Figure **Performance of the proposed methods with related parameters of three network construction strategies on recommendation retrieval criteria**. (A–C) Recall enhancement. (D–F) Hit-rate at *L* = 20. Results are obtained by 10-fold cross-validation experiments on Netflix (4555 objects and 5000 users) with cosine similarity measure. Restart probabilities for random walk approaches are set to 0.9. The higher the recall enhancement, the better the recommendation retrieval performance. The higher the hit-rate at *L* = 20, the better the retrieval performance.
(TIF)

S7 Figure **Performance of the proposed methods with related parameters of three network construction strategies on recommendation diversity criteria**. (A–C) Mean personalization. (D–F) Mean novelty. Results are obtained by 10-fold cross-validation experiments on Netflix (4555 objects and 5000 users) with cosine similarity measure. Restart probabilities for random walk approaches are set to 0.9. The higher the mean personalization, the better the recommendation diversity performance. The higher the mean novelty, the better the diversity performance.
(TIF)

S8 Figure **Performance of the proposed methods with related parameters of three network construction strategies on recommendation accuracy criteria**. (A–C) Mean relative rank. (D–F) Precision at *L* = 20. Results are obtained by 10-fold cross-validation experiments on Netflix (4555 objects and 5000 users) with Jaccard index measure. Restart probabilities for random walk approaches are set to 0.9. The lower the mean relative rank, the better the performance of recommendation accuracy. The higher the precision at *L* = 20, the better the recommendation accuracy.
(TIF)

S9 Figure **Performance of the proposed methods with related parameters of three network construction strategies on recommendation retrieval criteria**. (A–C) Recall enhancement. (D–F) Hit-rate at *L* = 20. Results are obtained by 10-fold cross-validation experiments on Netflix (4555 objects and 5000 users) with Jaccard index. Restart probabilities for random walk approaches are set to 0.9. The higher the recall enhancement, the better the recommendation retrieval performance. The higher the hit-rate at *L* = 20, the better the retrieval performance.
(TIF)

S10 Figure **Performance of the proposed methods with related parameters of three network construction strategies on recommendation diversity criteria**. (A–C) Mean personalization. (D–F) Mean novelty. Results are obtained by 10-fold cross-validation experiments on Netflix (4555 objects and 5000 users) with Jaccard index. Restart probabilities for random walk approaches are set to 0.9. The higher the mean personalization, the better the recommendation diversity performance. The higher the mean novelty, the better the diversity performance.
(TIF)

S1 Table **Performance of different methods.** Results are mean (standard derivation) obtained by 10-fold cross-validation experiments on MovieLens (5,000 users and 5,977 objects) with Jaccard index. Restart probabilities for random walk approaches are set to 0.9. *MRR* represents mean relative rank, *PR@20* represents precision at the default *L* value of 20, *RE* represents recall enhancement, *HR@20* represents hit-rate at *L* = 20, *MP* represents mean personalization, *MN* represents mean novelty.
(DOCX)

S2 Table **Performance of different methods.** Results are mean (standard derivation) obtained by 10-fold cross-validation experiments on Netflix (5,000 users and 4,555 objects) using cosine similarity measure. Restart probabilities for random walk approaches are set to 0.9. *MRR* represents mean relative rank, *PR@20* represents precision at the default *L* value of 20, *RE* represents recall enhancement, *HR@20* represents hit-rate at *L* = 20, *MP* represents mean personalization, *MN* represents mean novelty.
(DOCX)

S3 Table **Performance of different methods.** Results are mean (standard derivation) obtained by 10-fold cross-validation experiments on Netflix (5,000 users and 4,555 objects) using Jaccard index. Restart probabilities for random walk approaches are set to 0.9. *MRR* represents mean relative rank, *PR@20* represents precision at the default *L* value of 20, *RE* represents recall enhancement, *HR@20* represents hit-rate at *L* = 20, *MP* represents mean personalization, *MN* represents mean novelty.
(DOCX)

S4 Table **Performance of different methods.** Results are mean (standard derivation) obtained by 10-fold cross-validation experiments on MovieLens (9,757 users and 9,642 objects) using cosine similarity measure. Restart probabilities for random walk approaches are set to 0.9. *MRR* represents mean relative rank, *PR@20* represents precision at the default *L* value of 20, *RE* represents recall enhancement, *HR@20* represents hit-rate at *L* = 20, *MP* represents mean personalization, *MN* represents mean novelty.
(DOCX)

S5 Table **Performance of different methods.** Results are mean (standard derivation) obtained by 10-fold cross-validation experiments on MovieLens (9,757 users and 9,642 objects) using Jaccard index. Restart probabilities for random walk approaches are set to 0.9. *MRR* represents mean relative rank, *PR@20* represents precision at the default *L* value of 20, *RE* represents recall enhancement, *HR@20* represents hit-rate at *L* = 20, *MP* represents mean personalization, *MN* represents mean novelty.
(DOCX)

S1 Text **The text includes three parts as the motivations and intuitions behind the model, consistency between different similarity measures, and consistency between different data sets.**
(DOC)
