# Supplementary figures and images for: Walking on a User Similarity Network towards Personalized Recommendations
Source: PLoS One. 2014 Dec 9;9(12):e114662. doi: 10.1371/journal.pone.0114662 (PMC4260921; doi:10.1371/journal.pone.0114662)

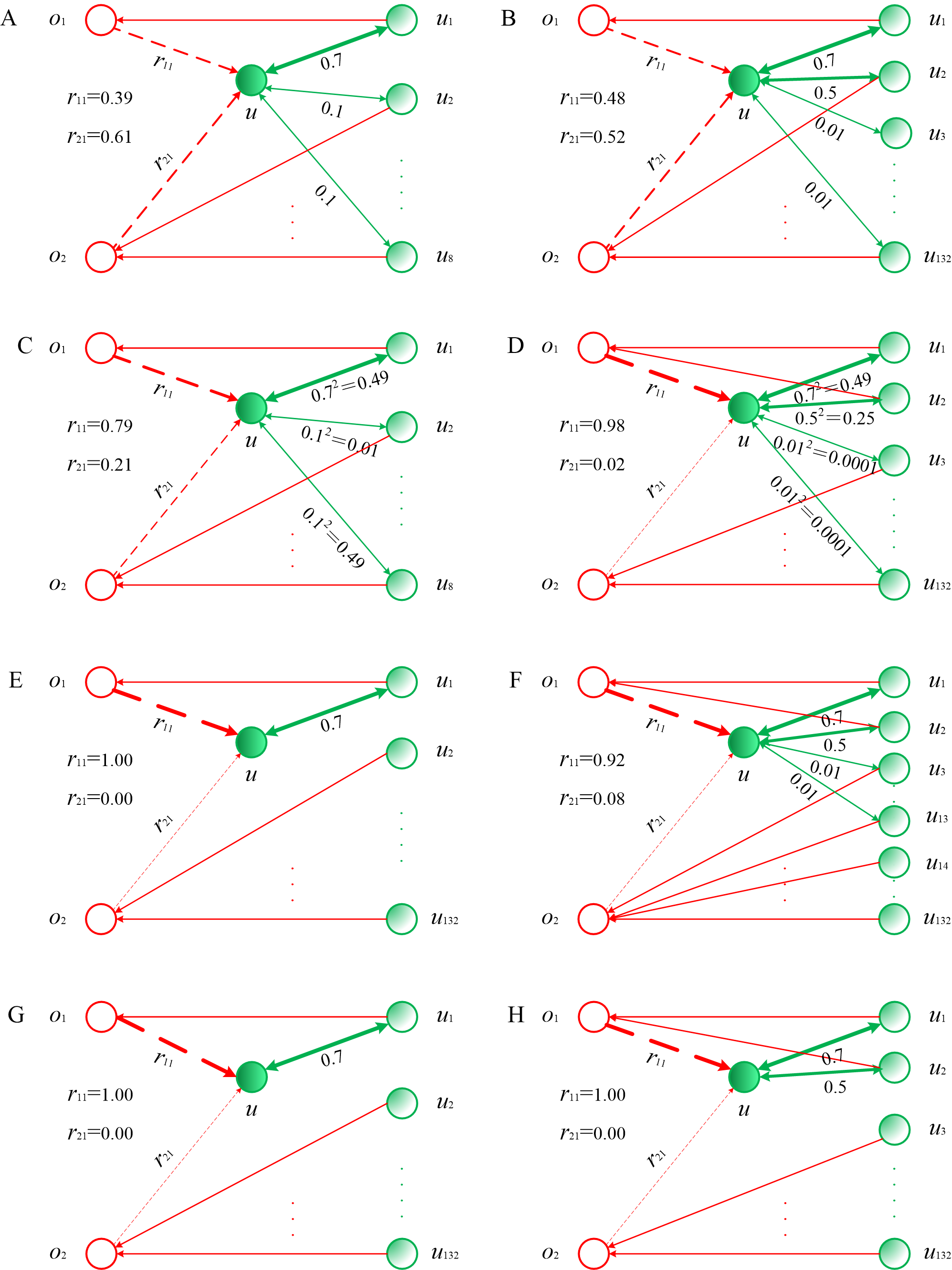

Supplement: S1 Figure — Effects of power law adjustment, nearest neighbor construction and threshold filtering to user similarity scores. A: Two objects o 1 and o 2 are assigned equal scores by random walk with restart probability at 0.9. B: o 1 is assigned a smaller score than o 2 by random walk with restart probability at 0.9. C: In contrast to (A), o 1 is assigned a larger score than o 2 after applying power-law adjustment (ß = 2) to user similarity scores. D: In contrast to (B), o 1 is assigned a larger score than o 2 after applying power-law adjustment (ß = 2) to user similarity scores. E: In contrast to (A), o 1 is assigned a larger score than o 2 after applying nearest neighbor construction (λ = 10%) to user similarity scores. F: In contrast to (B), o 1 is assigned a larger score than o 2 after applying nearest neighbor construction (λ = 10%) to user similarity scores. G: In contrast to (A), o 1 is assigned a larger score than o 2 after applying threshold filtering (δ = 0.20) to user similarity scores. H: In contrast to (B), o 1 is assigned a larger score than o 2 after applying threshold filtering (δ = 0.20) to user similarity scores. (TIF) [file pone.0114662.s001.tif]

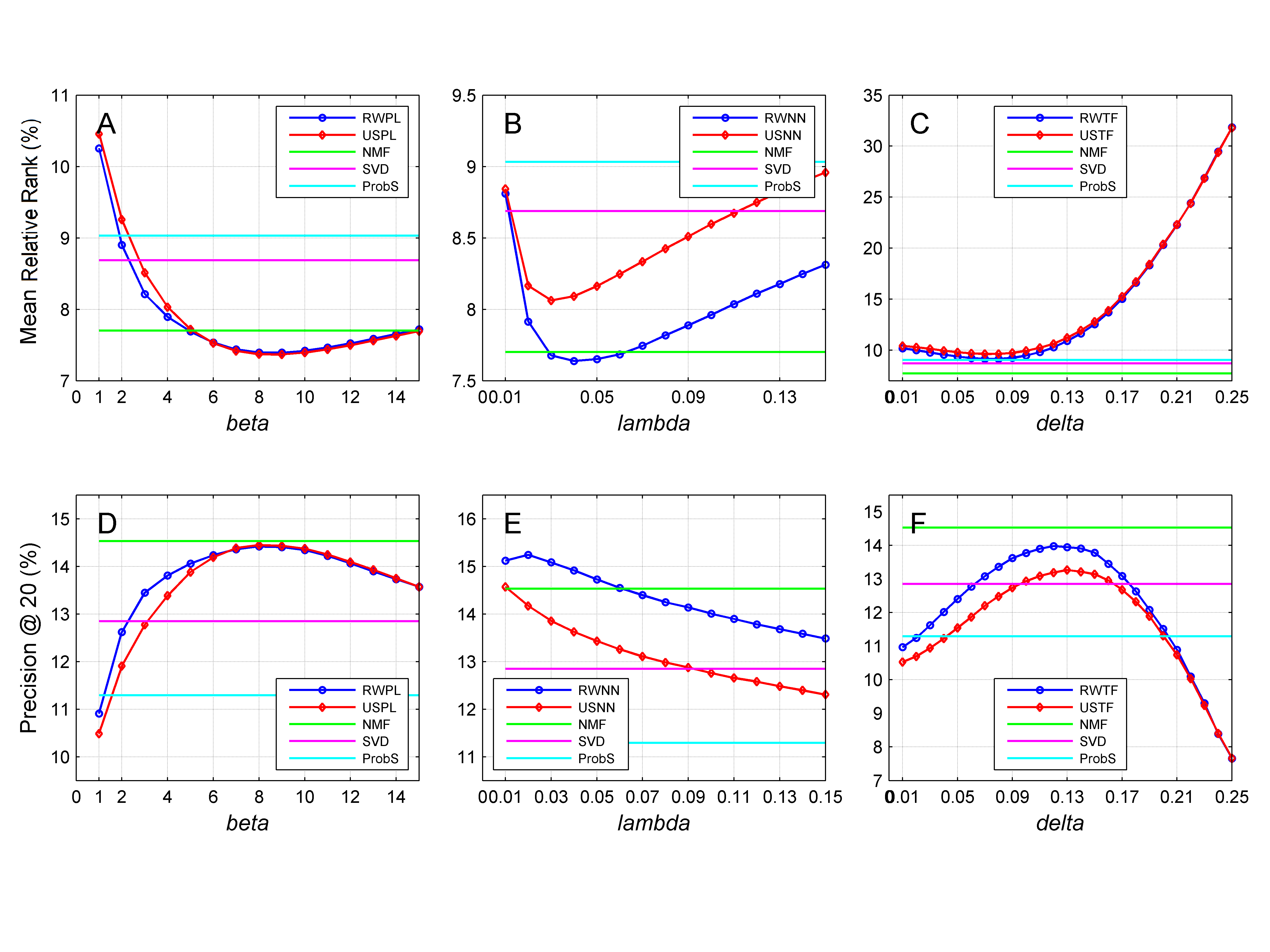

Supplement: S2 Figure — Performance of the proposed methods with related parameters of three network construction strategies on recommendation accuracy criteria. (A–C) Mean relative rank. (D–F) Precision at L = 20. Results are obtained by 10-fold cross-validation experiments on MovieLens (5,000 users and 5,977 objects) with the Jaccard index measure. Restart probabilities for random walk approaches are set to 0.9. The lower the mean relative rank, the better the performance of recommendation accuracy. The higher the precision at L = 20, the better the recommendation accuracy. (TIF) [file pone.0114662.s002.tif]

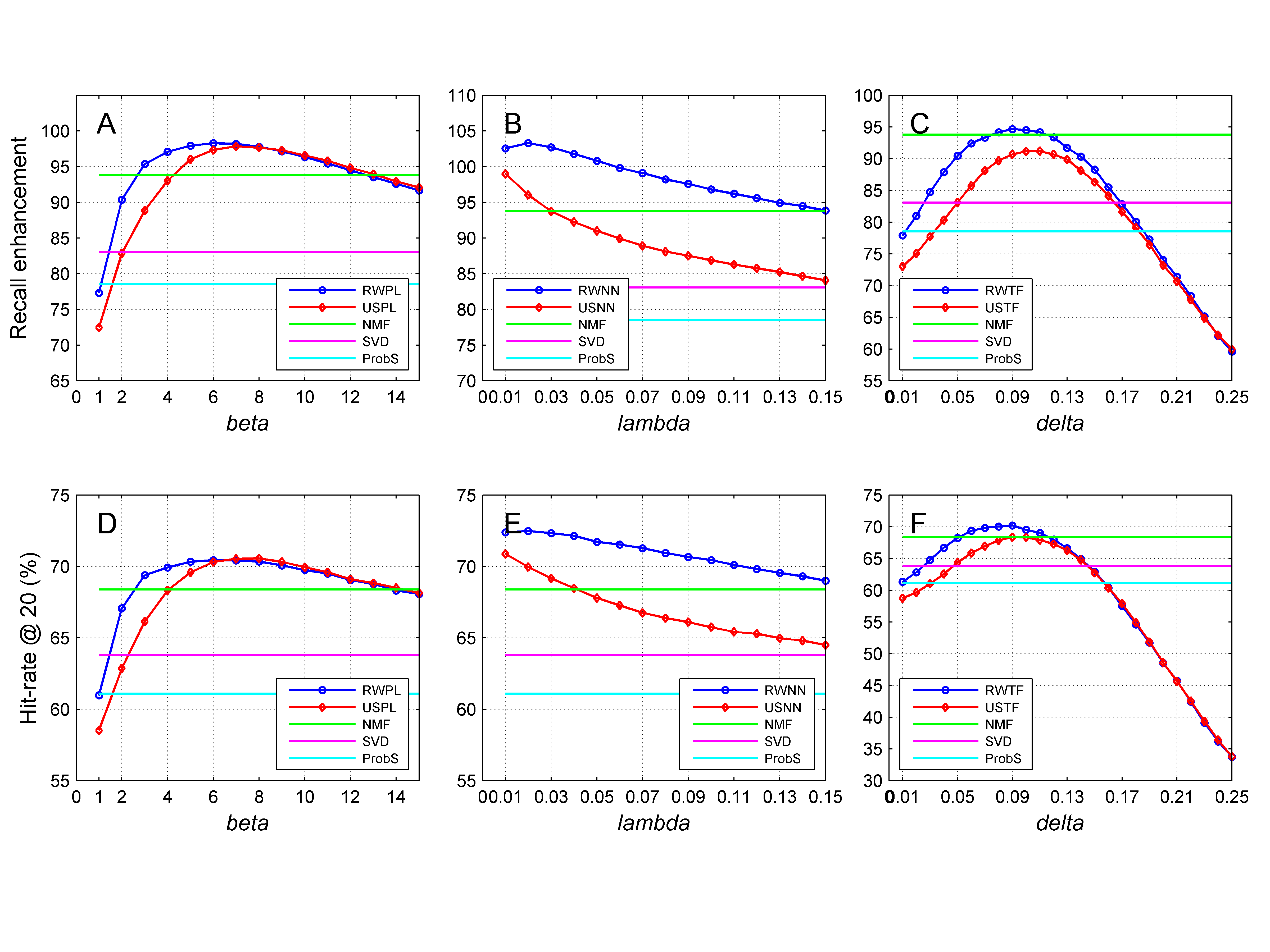

Supplement: S3 Figure — Performance of the proposed methods with related parameters of three network construction strategies on recommendation retrieval criteria. (A–C) Recall enhancement. (D–F) Hit-rate at L = 20. Results are obtained by 10-fold cross-validation experiments on MovieLens (5977 objects and 5000 users) with Jaccard index. Restart probabilities for random walk approaches are set to 0.9. The higher the recall enhancement, the better the recommendation retrieval performance. The higher the hit-rate at L = 20, the better the retrieval performance. (TIF) [file pone.0114662.s003.tif]

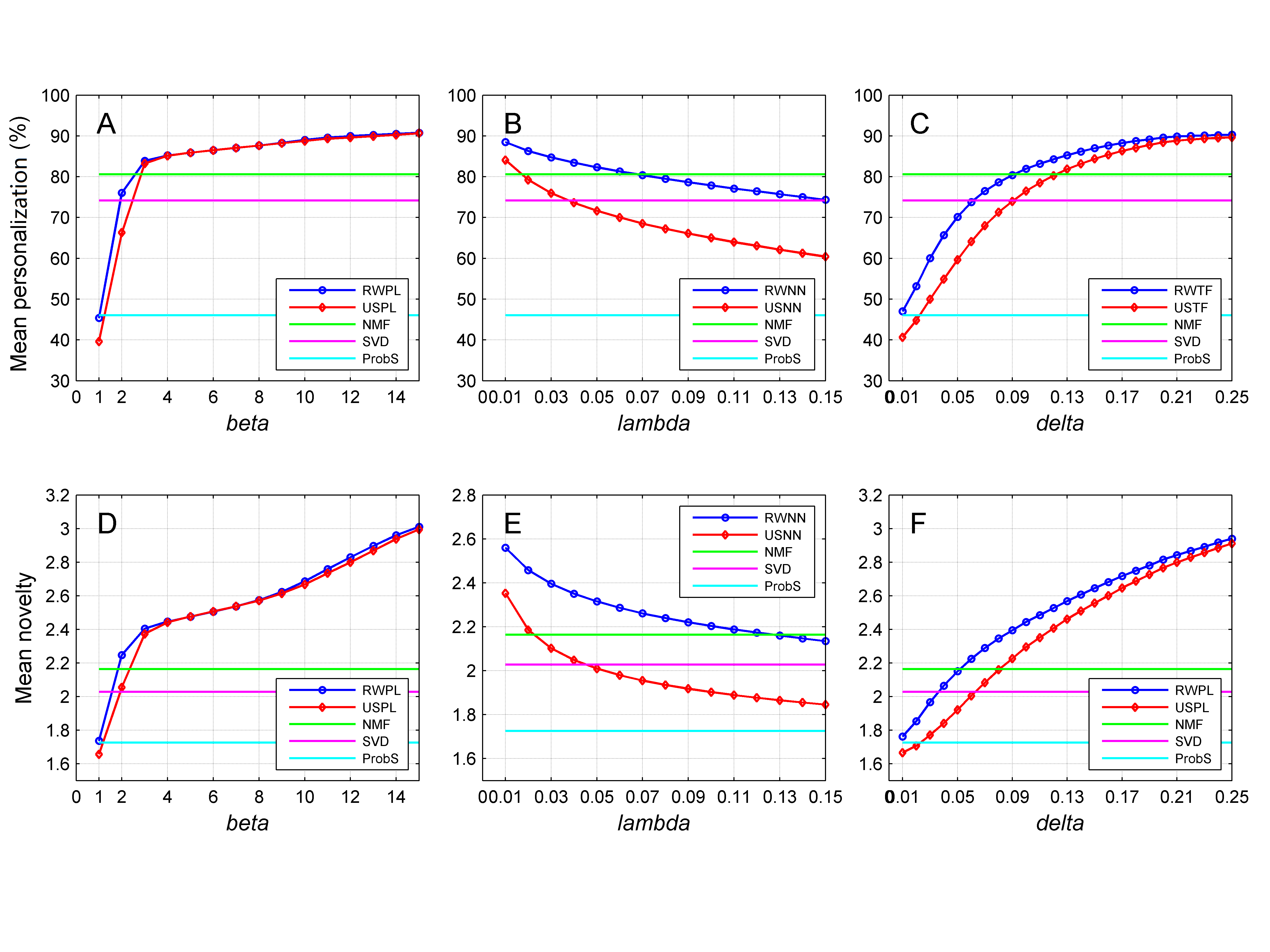

Supplement: S4 Figure — Performance of the proposed methods with related parameters of three network construction strategies on recommendation diversity criteria. (A–C) Mean personalization. (D–F) Mean novelty. Results are obtained by 10-fold cross-validation experiments on MovieLens (5977 objects and 5000 users) with Jaccard index. Restart probabilities for random walk approaches are set to 0.9. The higher the mean personalization, the better the recommendation diversity performance. The higher the mean novelty, the better the diversity performance. (TIF) [file pone.0114662.s004.tif]

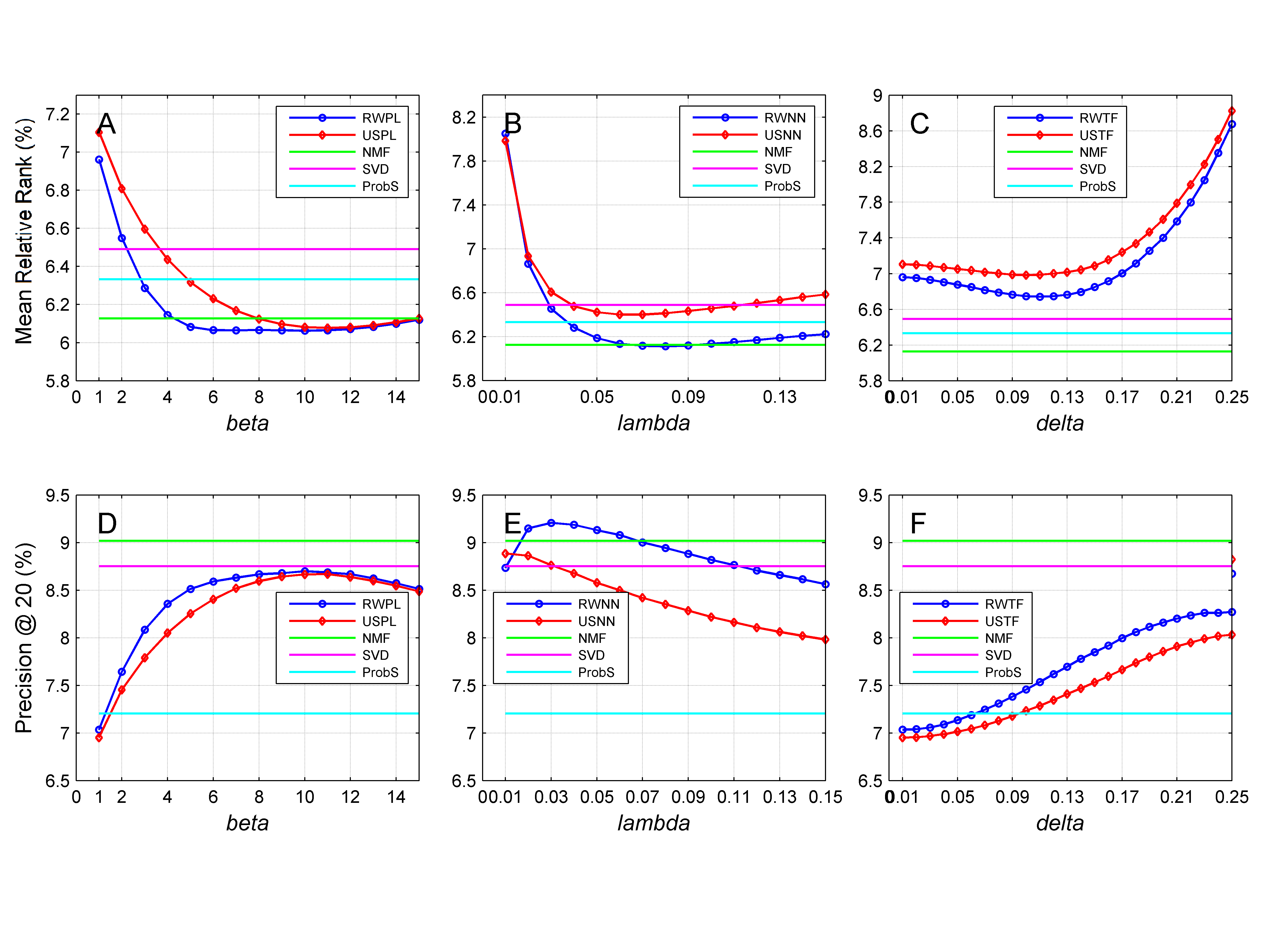

Supplement: S5 Figure — Performance of the proposed methods with related parameters of three network construction strategies on recommendation accuracy criteria. (A–C) Mean relative rank. (D–F) Precision at L = 20. Results are obtained by 10-fold cross-validation experiments on Netflix (4555 objects and 5000 users) with cosine similarity measure. Restart probabilities for random walk approaches are set to 0.9. The lower the mean relative rank, the better the performance of recommendation accuracy. The higher the precision at L = 20, the better the recommendation accuracy. (TIF) [file pone.0114662.s005.tif]

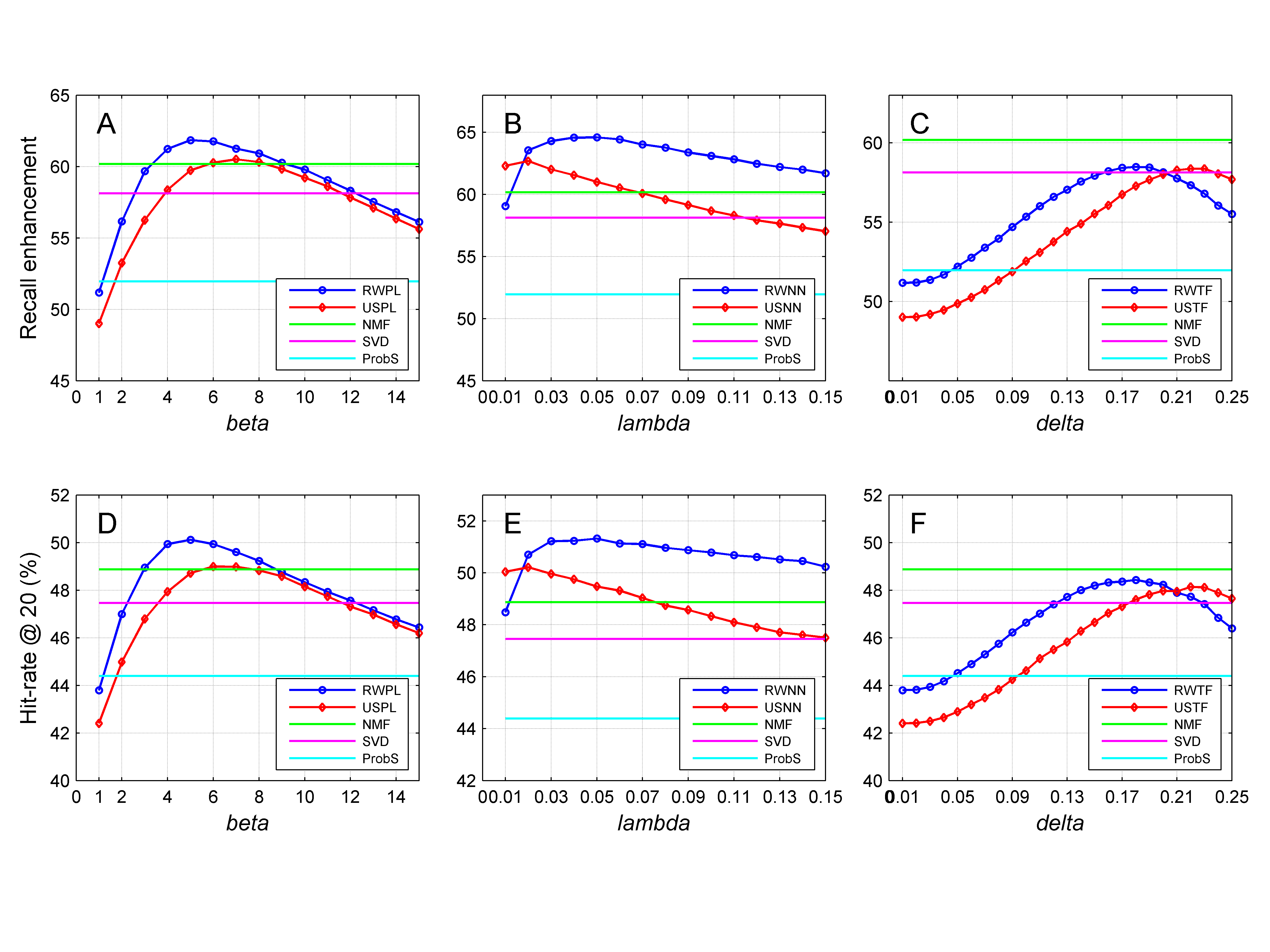

Supplement: S6 Figure — Performance of the proposed methods with related parameters of three network construction strategies on recommendation retrieval criteria. (A–C) Recall enhancement. (D–F) Hit-rate at L = 20. Results are obtained by 10-fold cross-validation experiments on Netflix (4555 objects and 5000 users) with cosine similarity measure. Restart probabilities for random walk approaches are set to 0.9. The higher the recall enhancement, the better the recommendation retrieval performance. The higher the hit-rate at L = 20, the better the retrieval performance. (TIF) [file pone.0114662.s006.tif]

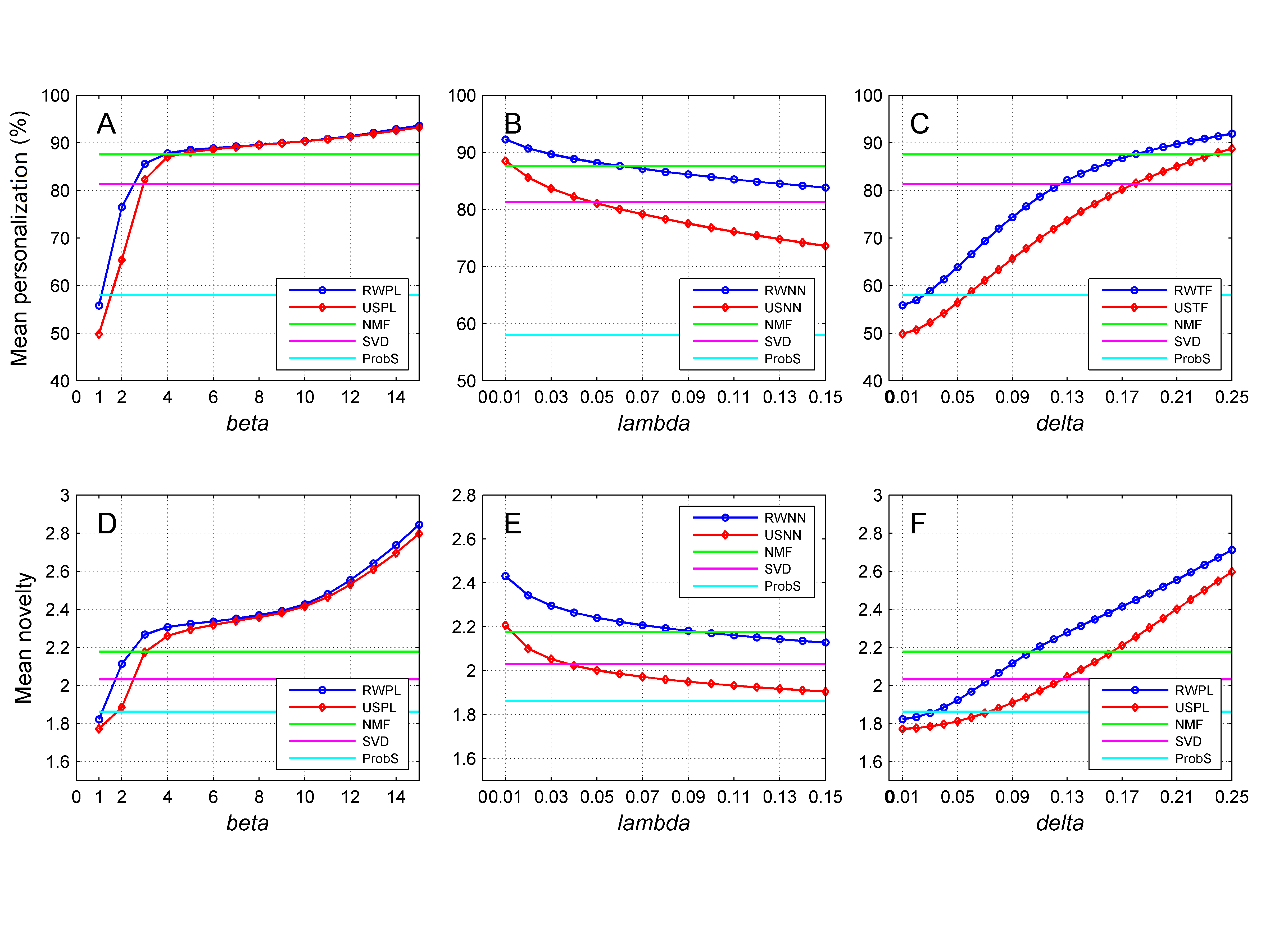

Supplement: S7 Figure — Performance of the proposed methods with related parameters of three network construction strategies on recommendation diversity criteria. (A–C) Mean personalization. (D–F) Mean novelty. Results are obtained by 10-fold cross-validation experiments on Netflix (4555 objects and 5000 users) with cosine similarity measure. Restart probabilities for random walk approaches are set to 0.9. The higher the mean personalization, the better the recommendation diversity performance. The higher the mean novelty, the better the diversity performance. (TIF) [file pone.0114662.s007.tif]

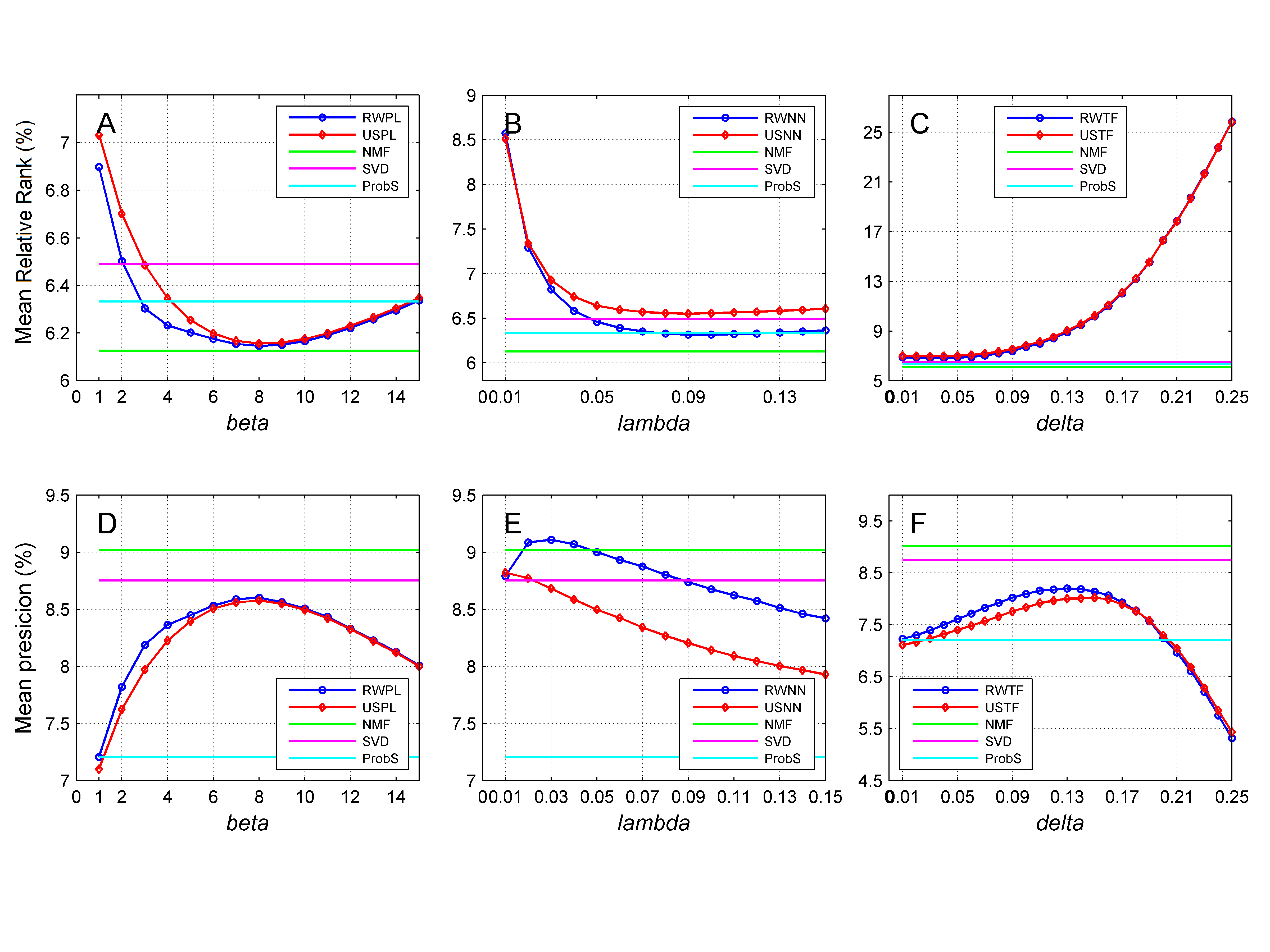

Supplement: S8 Figure — Performance of the proposed methods with related parameters of three network construction strategies on recommendation accuracy criteria. (A–C) Mean relative rank. (D–F) Precision at L = 20. Results are obtained by 10-fold cross-validation experiments on Netflix (4555 objects and 5000 users) with Jaccard index measure. Restart probabilities for random walk approaches are set to 0.9. The lower the mean relative rank, the better the performance of recommendation accuracy. The higher the precision at L = 20, the better the recommendation accuracy. (TIF) [file pone.0114662.s008.tif]

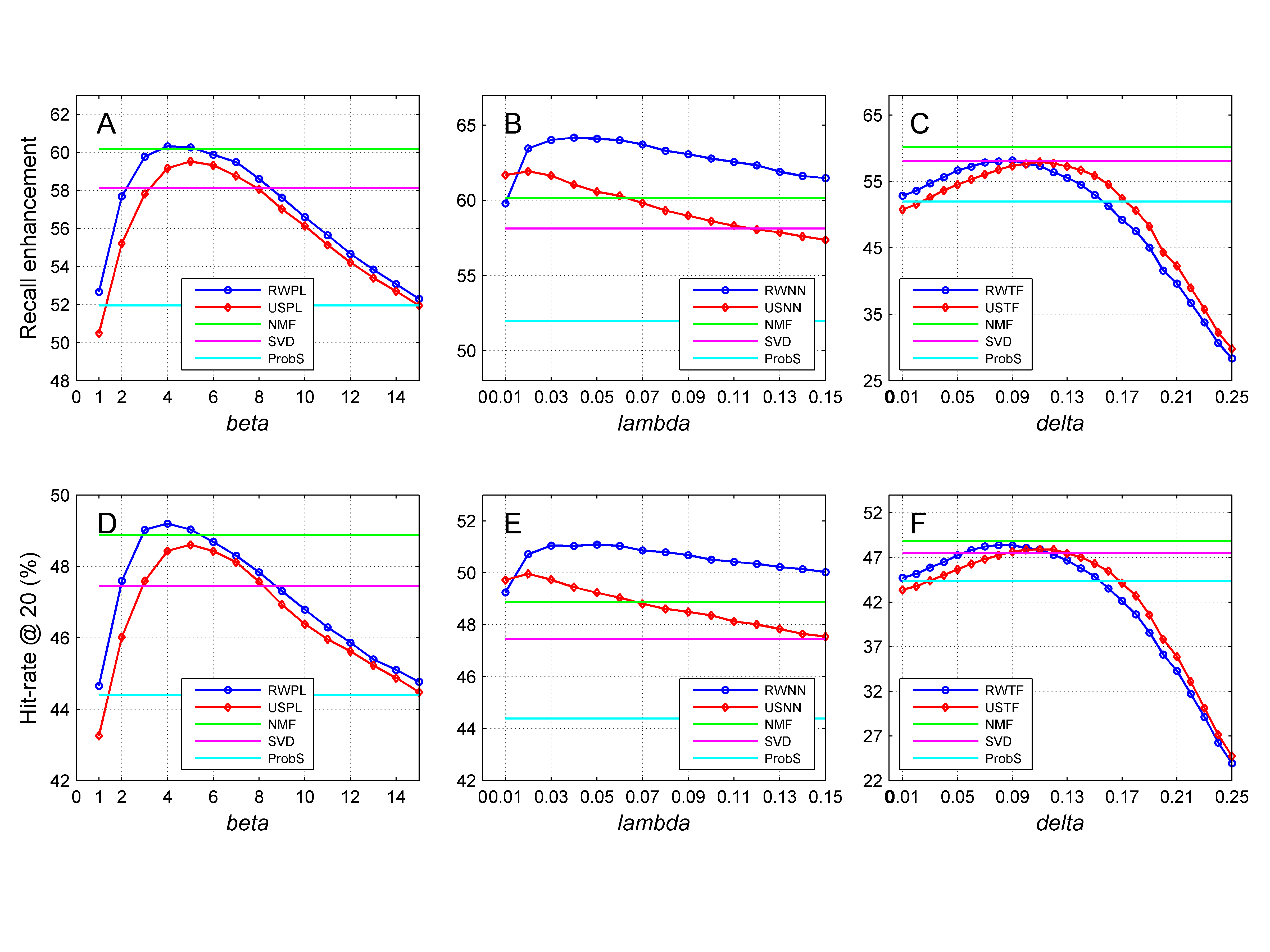

Supplement: S9 Figure — Performance of the proposed methods with related parameters of three network construction strategies on recommendation retrieval criteria. (A–C) Recall enhancement. (D–F) Hit-rate at L = 20. Results are obtained by 10-fold cross-validation experiments on Netflix (4555 objects and 5000 users) with Jaccard index. Restart probabilities for random walk approaches are set to 0.9. The higher the recall enhancement, the better the recommendation retrieval performance. The higher the hit-rate at L = 20, the better the retrieval performance. (TIF) [file pone.0114662.s009.tif]

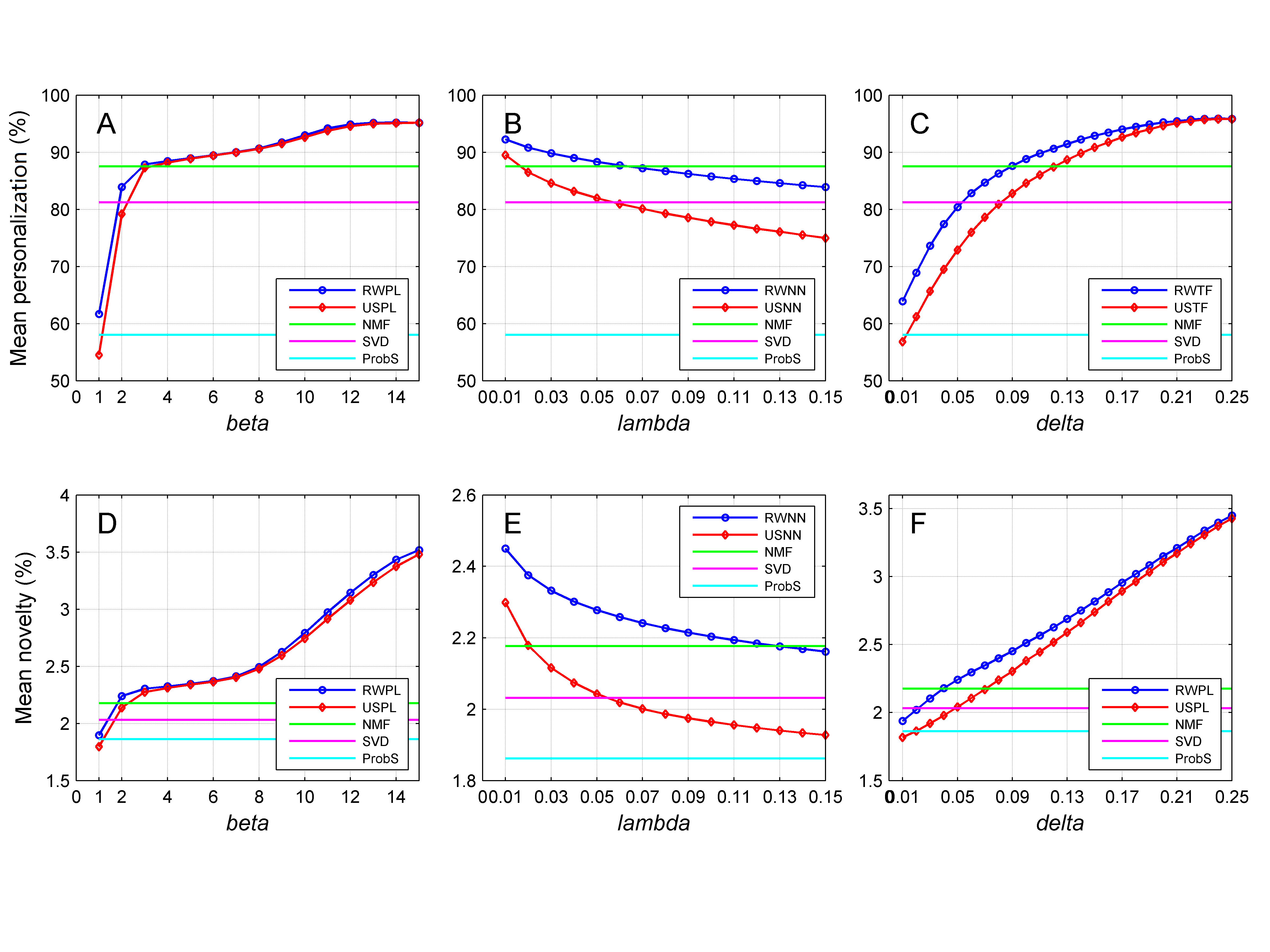

Supplement: S10 Figure — Performance of the proposed methods with related parameters of three network construction strategies on recommendation diversity criteria. (A–C) Mean personalization. (D–F) Mean novelty. Results are obtained by 10-fold cross-validation experiments on Netflix (4555 objects and 5000 users) with Jaccard index. Restart probabilities for random walk approaches are set to 0.9. The higher the mean personalization, the better the recommendation diversity performance. The higher the mean novelty, the better the diversity performance. (TIF) [file pone.0114662.s010.tif]
