# Supplementary material for: Walking on a User Similarity Network towards Personalized Recommendations
Source: PLoS One. 2014 Dec 9;9(12):e114662. doi: 10.1371/journal.pone.0114662 (PMC4260921; doi:10.1371/journal.pone.0114662)
Supplement: S1 Table — Performance of different methods. Results are mean (standard derivation) obtained by 10-fold cross-validation experiments on MovieLens (5,000 users and 5,977 objects) with Jaccard index. Restart probabilities for random walk approaches are set to 0.9. MRR represents mean relative rank, PR@20 represents precision at the default L value of 20, RE represents recall enhancement, HR@20 represents hit-rate at L = 20, MP represents mean personalization, MN represents mean novelty. (DOCX) [file pone.0114662.s011.docx]

**Table S1.** **Performance of different methods.** Results are mean (standard derivation) obtained by 10-fold cross-validation experiments on MovieLens (5,000 users and 5,977 objects) with Jaccard index. Restart probabilities for random walk approaches are set to 0.9. *MRR* represents mean relative rank, *PR@20* represents precision at the default *L* value of 20, *RE* represents recall enhancement, *HR@20* represents hit-rate at *L* = 20, *MP* represents mean personalization, *MN* represents mean novelty.

| **Method** | *MRR* (%) | *PR*@20 (%) | *RE* | *HR*@20 (%) | *MP* (%) | *MN* |
| --- | --- | --- | --- | --- | --- | --- |
| RWPL (*ß* = 9) | **7.39 (0.05)** | 14.41 (0.07) | 97.14 (0.58) | 70.07 (0.43) | **88.30 (0.22)** | **2.62 (0.04)** |
| RWNN (*λ* = 0.02) | 7.91 (0.04) | **15.24 (0.08)** | **103.30 (0.45)** | **72.48 (0.37)** | 86.31 (0.23) | 2.46 (0.03) |
| RWTF (*δ* = 0.07) | 9.15 (0.04) | 13.08 (0.06) | 93.32 (0.57) | 69.82 (0.48) | 76.51 (0.24) | 2.29 (0.04) |
| USPL (*ß* = 9) | 7.40 (0.08) | 14.43 (0.09) | 97.28 (0.73) | 70.32 (0.45) | 88.21 (0.21) | 2.61 (0.05) |
| USNN (*λ* = 0.01) | 8.84 (0.07) | 14.57 (0.11) | 98.99 (0.81) | 70.87 (0.37) | 84.08 (0.23) | 2.35 (0.09) |
| USTF (*δ* = 0.07) | 9.62 (0.08) | 12.21 (0.10) | 88.11 (0.75) | 66.93 (0.42) | 68.00 (0.22) | 2.08 (0.15) |
| NMF | 7.70 (0.08) | 14.53 (0.06) | 93.78 (0.67) | 68.39 (0.48) | 80.57 (0.20) | 2.16 (0.08) |
| SVD | 8.69 (0.09) | 12.85 (0.05) | 83.06 (0.53) | 63.77 (0.37) | 74.16 (0.17) | 2.03 (0.05) |
| ProbS | 9.03 (0.07) | 11.29 (0.07) | 78.51 (0.67) | 61.09 (0.27) | 46.05 (0.25) | 1.73 (0.13) |
