# Supplementary material for: Walking on a User Similarity Network towards Personalized Recommendations
Source: PLoS One. 2014 Dec 9;9(12):e114662. doi: 10.1371/journal.pone.0114662 (PMC4260921; doi:10.1371/journal.pone.0114662)
Supplement: S2 Table — Performance of different methods. Results are mean (standard derivation) obtained by 10-fold cross-validation experiments on Netflix (5,000 users and 4,555 objects) using cosine similarity measure. Restart probabilities for random walk approaches are set to 0.9. MRR represents mean relative rank, PR@20 represents precision at the default L value of 20, RE represents recall enhancement, HR@20 represents hit-rate at L = 20, MP represents mean personalization, MN represents mean novelty. (DOCX) [file pone.0114662.s012.docx]

**Table S2. Performance of different methods.** Results are mean (standard derivation) obtained by 10-fold cross-validation experiments on Netflix (5,000 users and 4,555 objects) using cosine similarity measure. Restart probabilities for random walk approaches are set to 0.9. *MRR* represents mean relative rank, *PR@20* represents precision at the default *L* value of 20, *RE* represents recall enhancement, *HR@20* represents hit-rate at *L* = 20, *MP* represents mean personalization, *MN* represents mean novelty.

| **Method** | *MRR* (%) | *PR*@20 (%) | *RE* | *HR*@20 (%) | *MP* (%) | *MN* |
| --- | --- | --- | --- | --- | --- | --- |
| RWPL (*ß* = 6) | **6.07 (0.05)** | 8.59 (0.07) | 61.76 (0.65) | 49.94 (0.41) | **90.34 (0.22)** | **2.48 (0.04)** |
| RWNN (*λ* = 0.05) | 6.19 (0.05) | **9.13 (0.06)** | **64.60 (0.36)** | **51.32 (0.48)** | 89.64 (0.22) | 2.34 (0.03) |
| RWTF (*δ* = 0.11) | 6.74 (0.04) | 7.54 (0.05) | 56.01 (0.53) | 47.01 (0.48) | 87.62 (0.26) | 2.45 (0.05) |
| USPL (*ß* = 10) | 6.08 (0.08) | 8.67 (0.11) | 59.22 (0.79) | 48.15 (0.51) | 90.31 (0.22) | 2.45 (0.03) |
| USNN (*λ* = 0.08) | 6.40 (0.08) | 8.50 (0.11) | 60.53 (0.84) | 49.32 (0.33) | 83.63 (0.21) | 2.10 (0.10) |
| USTF (*δ* = 0.10) | 6.98 (0.08) | 7.23 (0.09) | 52.54 (0.77) | 44.62 (0.35) | 81.52 (0.24) | 2.25 (0.17) |
| NMF | 6.13 (0.09) | 9.02 (0.07) | 60.17 (0.63) | 48.87 (0.48) | 87.51 (0.28) | 2.18 (0.09) |
| SVD | 6.49 (0.06) | 8.75 (0.07) | 58.13 (0.51) | 47.46 (0.38) | 81.22 (0.15) | 2.03 (0.08) |
| ProbS | 6.33 (0.07) | 7.20 (0.08) | 51.96 (0.64) | 44.39 (0.28) | 58.04 (0.23) | 1.86 (0.16) |
