# Supplementary material for: Walking on a User Similarity Network towards Personalized Recommendations
Source: PLoS One. 2014 Dec 9;9(12):e114662. doi: 10.1371/journal.pone.0114662 (PMC4260921; doi:10.1371/journal.pone.0114662)
Supplement: S3 Table — Performance of different methods. Results are mean (standard derivation) obtained by 10-fold cross-validation experiments on Netflix (5,000 users and 4,555 objects) using Jaccard index. Restart probabilities for random walk approaches are set to 0.9. MRR represents mean relative rank, PR@20 represents precision at the default L value of 20, RE represents recall enhancement, HR@20 represents hit-rate at L = 20, MP represents mean personalization, MN represents mean novelty. (DOCX) [file pone.0114662.s013.docx]

**Table S3. Performance of different methods.** Results are mean (standard derivation) obtained by 10-fold cross-validation experiments on Netflix (5,000 users and 4,555 objects) using Jaccard index. Restart probabilities for random walk approaches are set to 0.9. *MRR* represents mean relative rank, *PR@20* represents precision at the default *L* value of 20, *RE* represents recall enhancement, *HR@20* represents hit-rate at *L* = 20, *MP* represents mean personalization, *MN* represents mean novelty.

| **Method** | *MRR* (%) | *PR*@20 (%) | *RE* | *HR*@20 (%) | *MP* (%) | *MN* |
| --- | --- | --- | --- | --- | --- | --- |
| RWPL (*ß* = 8) | 6.15 (0.05) | 8.60 (0.06) | 58.60 (0.49) | 47.83 (0.44) | **94.17** (0.21) | **2.97** (0.03) |
| RWNN (*λ* = 0.11) | 6.32 (0.05) | 8.74 (0.05) | **63.07 (0.42)** | **50.68 (0.37)** | 89.82 (0.22) | 2.34 (0.03) |
| RWTF (*δ* = 0.03) | 6.83 (0.05) | 7.50 (0.04) | 55.66 (0.56) | 46.48 (0.44) | 92.88 (0.26) | 2.82 (0.06) |
| USPL (*ß* = 8) | 6.16 (0.08) | 8.58 (0.12) | 58.06 (0.84) | 47.57 (0.51) | 93.77 (0.23) | 2.91 (0.03) |
| USNN (*λ* = 0.11) | 6.55 (0.07) | 8.20 (0.09) | 58.98 (0.74) | 48.49 (0.36) | 84.61 (0.22) | 2.10 (0.11) |
| USTF (*δ* = 0.03) | 6.98 (0.07) | 7.23 (0.10) | 52.60 (0.69) | 44.39 (0.44) | 90.85 (0.24) | 2.74 (0.16) |
| NMF | **6.13 (0.09)** | **9.02 (0.07)** | 60.17 (0.63) | 48.87 (0.48) | 87.51 (0.28) | 2.18 (0.09) |
| SVD | 6.49 (0.06) | 8.75 (0.07) | 58.13 (0.51) | 47.46 (0.38) | 81.22 (0.15) | 2.03 (0.08) |
| ProbS | 6.33 (0.07) | 7.20 (0.08) | 51.96 (0.64) | 44.39 (0.28) | 58.04 (0.23) | 1.86 (0.16) |
