# Supplementary material for: Walking on a User Similarity Network towards Personalized Recommendations
Source: PLoS One. 2014 Dec 9;9(12):e114662. doi: 10.1371/journal.pone.0114662 (PMC4260921; doi:10.1371/journal.pone.0114662)
Supplement: S4 Table — Performance of different methods. Results are mean (standard derivation) obtained by 10-fold cross-validation experiments on MovieLens (9,757 users and 9,642 objects) using cosine similarity measure. Restart probabilities for random walk approaches are set to 0.9. MRR represents mean relative rank, PR@20 represents precision at the default L value of 20, RE represents recall enhancement, HR@20 represents hit-rate at L = 20, MP represents mean personalization, MN represents mean novelty. (DOCX) [file pone.0114662.s014.docx]

**Table S4.** **Performance of different methods.** Results are mean (standard derivation) obtained by 10-fold cross-validation experiments on MovieLens (9,757 users and 9,642 objects) using cosine similarity measure. Restart probabilities for random walk approaches are set to 0.9. *MRR* represents mean relative rank, *PR@20* represents precision at the default *L* value of 20, *RE* represents recall enhancement, *HR@20* represents hit-rate at *L* = 20, *MP* represents mean personalization, *MN* represents mean novelty.

| **Method** | *MRR* (%) | *PR*@20 (%) | *RE* | *HR*@20 (%) | *MP* (%) | *MN* |
| --- | --- | --- | --- | --- | --- | --- |
| RWPL (*ß* = 12) | **4.82 (0.02)** | 15.07 (0.08) | 164.73 (1.85) | 71.16 (0.63) | 88.12 (0.11) | 2.68 (0.01) |
| RWNN (*λ* = 0.02) | 4.95 (0.02) | **15.72 (0.07)** | **170.76 (1.78)** | **73.18 (0.57)** | 88.00 (0.13) | 2.92 (0.03) |
| RWTF (*δ* = 0.16) | 5.96 (0.01) | 13.63 (0.06) | 154.80 (1.98) | 69.64 (0.58) | 86.83 (0.14) | 3.26 (0.04) |
| USPL (*ß* = 12) | 4.83 (0.02) | 14.96 (0.08) | 163.49 (1.58) | 63.41 (0.62) | 89.24 (0.11) | 2.67 (0.01) |
| USNN (*λ* = 0.02) | 5.25 (0.01) | 14.40 (0.06) | 156.53 (1.62) | 60.80 (0.58) | 80.86 (0.14) | 3.29 (0.05) |
| USTF (*δ* = 0.16) | 6.52 (0.01) | 12.34 (0.04) | 145.34 (1.44) | 67.42 (0.68) | 84.98 (0.12) | 3.55 (0.07) |
| NMF | 5.27 (0.03) | 14.60 (0.14) | 151.72 (1.27) | 68.11 (0.28) | **90.15 (0.44)** | 3.85 (0.12) |
| SVD | 5.94 (0.04) | 12.79 (0.07) | 131.39 (1.35) | 63.41 (0.42) | 89.24 (0.17) | **4.21 (0.03)** |
| ProbS | 6.13 (0.01) | 11.27 (0.03) | 128.54 (1.42) | 60.80 (0.55) | 80.86 (0.12) | 3.82 (0.09) |
