# Supplementary material for: Walking on a User Similarity Network towards Personalized Recommendations
Source: PLoS One. 2014 Dec 9;9(12):e114662. doi: 10.1371/journal.pone.0114662 (PMC4260921; doi:10.1371/journal.pone.0114662)
Supplement: S5 Table — Performance of different methods. Results are mean (standard derivation) obtained by 10-fold cross-validation experiments on MovieLens (9,757 users and 9,642 objects) using Jaccard index. Restart probabilities for random walk approaches are set to 0.9. MRR represents mean relative rank, PR@20 represents precision at the default L value of 20, RE represents recall enhancement, HR@20 represents hit-rate at L = 20, MP represents mean personalization, MN represents mean novelty. (DOCX) [file pone.0114662.s015.docx]

**Table S5.** **Performance of different methods.** Results are mean (standard derivation) obtained by 10-fold cross-validation experiments on MovieLens (9,757 users and 9,642 objects) using Jaccard index. Restart probabilities for random walk approaches are set to 0.9. *MRR* represents mean relative rank, *PR@20* represents precision at the default *L* value of 20, *RE* represents recall enhancement, *HR@20* represents hit-rate at *L* = 20, *MP* represents mean personalization, *MN* represents mean novelty.

| **Method** | *MRR* (%) | *PR*@20 (%) | *RE* | *HR*@20 (%) | *MP* (%) | *MN* |
| --- | --- | --- | --- | --- | --- | --- |
| RWPL (*ß* = 10) | **4.92 (0.02)** | **14.84 (0.12)** | 162.56 (1.88) | 71.00 (0.61) | 88.01 (0.17) | 2.68 (0.01) |
| RWNN (*λ* = 0.04) | 5.14 (0.02) | 14.96 (0.09) | **165.26 (1.87)** | **72.04 (0.64)** | 87.06 (0.16) | 2.97 (0.03) |
| RWTF (*δ* = 0.07) | 6.27 (0.02) | 13.18 (0.09) | 154.47 (1.63) | 70.44 (0.61) | 85.56 (0.23) | 3.24 (0.04) |
| USPL (*ß* = 10) | 4.92 (0.02) | 14.81 (0.08) | 162.26 (1.60) | 70.79 (0.61) | 88.02 (0.11) | 2.68 (0.01) |
| USNN (*λ* = 0.04) | 5.51 (0.02) | 13.60 (0.08) | 149.46 (1.40) | 67.78 (0.60) | 85.85 (0.10) | 3.38 (0.06) |
| USTF (*δ* = 0.07) | 6.61 (0.01) | 12.11 (0.04) | 143.61 (1.34) | 66.65 (0.46) | 84.83 (0.12) | 3.58 (0.08) |
| NMF | 5.27 (0.03) | 14.60 (0.14) | 151.72 (1.27) | 68.11 (0.28) | **90.15 (0.44)** | **3.85 (0.12)** |
| SVD | 5.94 (0.04) | 12.79 (0.07) | 131.39 (1.35) | 63.41 (0.42) | 89.24 (0.17) | 4.21 (0.03) |
| ProbS | 6.13 (0.01) | 11.27 (0.03) | 128.54 (1.42) | 60.80 (0.55) | 80.86 (0.12) | 3.82 (0.09) |
