# Supplementary material for: Walking on a User Similarity Network towards Personalized Recommendations
Source: PLoS One. 2014 Dec 9;9(12):e114662. doi: 10.1371/journal.pone.0114662 (PMC4260921; doi:10.1371/journal.pone.0114662)
Supplement: S1 Text — The text includes three parts as the motivations and intuitions behind the model, consistency between different similarity measures, and consistency between different data sets. (DOC) [file pone.0114662.s016.doc]

Text S1 for

Walking on a User Similarity Network towards Personalized Recommendations

Mingxin Gan ^1,§^

^1^: Dongling School of Economics and Management
University of Science and Technology Beijing
Beijing, 100083, China

^§^: To whom correspondence should be addressed.
Email: [ganmx@ustb.edu.cn](mailto:ganmx@ustb.edu.cn)

# Motivations and intuitions behind the model

The basic assumption of many mainstream recommendation methods is that users having similar preferences in history would also have similar preferences in the future. Therefore, discriminant scores for candidate objects can be calculated as the weighted average of preferences of other users, with the weights being estimated using the historical data. Nevertheless, this formulation, though having been successfully applied to a number of situations, may fail in some special cases as illustrated in the following examples.

It is obvious that the similarity score between two users calculated as either cosine value or Jaccard index is proportional to the number of objects shared by the users. Therefore, a weak similarity score will be calculated for two users that share only a small number of objects. In the extreme case, a popular object must have been preferred by a large number of users, and thus the chance that two users both prefer this object will be high. As a result, it is likely that a weak similarity score will be assigned for two users simply because the two users share a popular object by chance. Such weak similarity scores will not be dominant in the downstream calculation of discriminant scores for candidate objects in most circumstances. However, in some cases, these weak similarity scores can adversely affect the calculation of discriminant scores and even lead to apparently wrong recommendations.

For example, in Figure S1 (A), a strong similarity score (0.7) has been calculated for two users *u* and *u*_1_, because they share a large fraction of common objects in historical data. Meanwhile, seven weak similarity scores (0.1) have been calculated for users *u* and *u_i_* (*i* = 2, …, 8), because *u* and *u_i_* (*i* = 2, …, 8) share only a small fraction of common objects in historical data. Now, for a candidate objects *o*_1_ that is preferred only by *u*_1_, a discriminant score of 0.39 is calculated according to the random walk method with restart probability equals 0.9. On the other hand, for another candidate objects *o*_2_ that is preferred by *u_i_* (*i* = 2, …, 8), a discriminant score of 0.61 is also calculated. Consequently, the rank of *o*_2_ will be higher to that of *o*_1_ in the recommendation list. However, by intuition, *u*_1_ should be considered more in the recommendation process, owing to the fact that *u*_1_ share a large fraction of objects with *u* in history, and thus these two users could be very likely to have similar preferences. In contrast, the other users *u_i_* (*i* = 2, …, 8) should be less considered, in the sense that the weak similarity scores between *u* and these users are likely to be obtained due to the share of some popular objects. Consequently, as a more reasonable recommendation, *o*_1_ should be qualified to occupy a place in front of *o*_2_ in the ranking list.

We further show in Figure S1 (B) a more comprehensive example that illustrates an obvious mistake introduced by a large number of weak similarity scores. Suppose that a strong similarity scores (0.7) have been calculated for *u* and *u*_1_ because they share a large fraction of common objects in historical data. Similarly, a strong similarity scores (0.5) have also been calculated for users *u* and *u*_2_. Meanwhile, 130 weak similarity scores (0.01) have been calculated for users *u* and *u_i_* (*i* = 3, …, 132), simply because *u* and *u_i_* (*i* = 3, …, 132) share only a small fraction of common objects in historical data. Now, for a candidate objects *o*_1_ that is preferred only by *u*_1_ and *u*_2_, a discriminant score of 0.48 is calculated according to random walk with restart probability at 0.9. On the other hand, for another candidate objects *o*_2_ that is preferred by *u_i_* (*i* = 3, …, 132), a discriminant score of 0.52 is calculated. Consequently, the rank of *o*_2_ will be higher than that of *o*_1_ in the recommendation list. However, by intuition, *u*_1_ and *u*_2_ are more important in the process of recommending objects for *u*, because each of them share a large fraction of objects with *u* in history and thus should have similar preferences with *u*. In contrast, the other users *u_i_* (*i* = 3,…,132) are less important when recommending objects for *u*, because the weak similarity scores between *u* and these users are likely to be obtained due to the share of some popular objects. Therefore, in a more reasonable recommendation, *o*_1_ should be qualified to rank in front of *o*_2_.

The influence of the parameter *ß* on the proposed method is illustrated in Figure S1 (C) and (D). The original similarity score for *u* and *u*_1_ is 0.7, and those for *u* and *u_i_* (*i* = 2, …, 8) are all 0.1. As shown in Figure S1 (C), when power-law function with exponent *ß* = 2 is applied, the user similarity score for *u* and *u*_1_ becomes 0.49, and those for *u* and *u_i_* (*i* = 2, …, 8) all become 0.01. As a result, the discriminant scores become 0.79 for *o*_1_ and 0.21 for *o*_2_, and thus *o*_1_ ranks much higher than *o*_2_. Similarly, the original similarity scores are 0.7 for *u* and *u*_1_, 0.5 for *u* and *u*_2_, and 0.01 for *u* and *u_i_* (*i* = 3, …, 132). As shown in Figure S1 (D), when power-law function with exponent *ß* = 2 is applied, the similarity scores become 0.49 for *u* and *u*_1_, 0.25 for *u* and *u*_2_, and 0.0001 for *u* and *u_i_* (*i* = 3, …, 132). Consequently, the discriminant scores become 0.98 for *o*_1_ and 0.02 for *o*_2_, and thus *o*_1_ ranks much higher than *o*_2_.

The influence of the parameter *λ* on the proposed method is illustrated in Figure S1 (E) and (F). The original similarity score for *u* and *u*_1_ is 0.7, and those for *u* and *u_i_* (*i* = 2, …, 8) are all 0.1. As shown in Figure S1 (E), when nearest neighbor function with *λ* = 10% is applied, the user similarity score for *u* and *u*_1_ remains 0.7, and those for *u* and *u_i_* (*i* = 2, …, 8) all become 0. As a result, the discriminant scores become 1.0 for *o*_1_ and 0 for *o*_2_. Similarly, in the original similarity scores are 0.7 for *u* and *u*_1_, 0.5 for *u* and *u*_2_, and 0.01 for *u* and *u_i_* (*i* = 3, …, 132). As shown in Figure S1 (F), when nearest neighbor function with *λ* = 10% is applied, the similarity scores remain 0.7 for *u* and *u*_1_, 0.5 for *u* and *u*_2_, and 0. 01 for *u* and *u_i_* (*i* = 3, …, 13), and 0 for *u* and *u_j_* (*j* = 14, …, 132). Consequently, the discriminant scores become 0.92 for *o*_1_ and 0.08 for *o*_2_, and thus *o*_1_ ranks much higher than *o*_2_.

The influence of the parameter *δ* on the proposed method is illustrated in Figure S1 (G) and (H). The original similarity score for *u* and *u*_1_ is 0.7, and those for *u* and *u_i_* (*i* = 2, …, 8) are all 0.1. As shown in Figure S1 (G), when threshold filtering function with *δ* = 0.20 is applied, the user similarity score for *u* and *u*_1_ remains 0.7, and those for *u* and *u_i_* (*i* = 2, …, 8) all become 0. As a result, the discriminant scores become 1.0 for *o*_1_ and 0 for *o*_2_. Similarly, the original similarity scores are 0.7 for *u* and *u*_1_, 0.5 for *u* and *u*_2_, and 0 for *u* and *u_i_* (*i* = 3, …, 132). As shown in Figure S1 (H), when threshold filtering function with *δ* = 0.20 is applied, the similarity scores remain 0.7 for *u* and *u*_1_, 0.5 for *u* and *u*_2_, and 0 for *u* and *u_i_* (*i* = 3, …, 132). Consequently, the discriminant scores become 1.0 for *o*_1_ and 0 for *o*_2_.

Based on the above analysis, we suppose that with suitable values for the exponent parameter, nearest neighbor parameter, or threshold filtering parameter, our method will effectively reduce the adverse influence of popular objects by magnifying the difference between strong similarities that mainly result from the share of a large fraction of objects between users and weak similarities that are mainly due to the share of a small number of popular objects between users.

# Consistency between different similarity measures

Although the cosine similarity measure has been widely used in the calculation of user similarity scores, there also exist several other methods for the same purpose. We therefore ask the question of whether the observed improvements in accuracy, retrieval and diversity criteria are consistent between different methods for calculating user similarity scores. To answer this question, we replaced cosine similarity with Jaccard index, repeated all the above experiments, and summarized the results in Table S1 and Figures S2, S3 and S4.

From the table and the figures, we observe the superior performance of the random walk with restart model over other methods. For example, when used with power-law adjusted network ($\beta=9$, $\gamma=0.9$), RWPL achieves a mean relative rank of 7.39%, a precision of 14.41%, a recall enhancement of 97.14, a hit-rate of 70.07%, a mean personality of 88.38%, and a mean novelty of 2.63. When used with nearest neighbor network ($\lambda=0.02$, $\gamma=0.9$), RWNN achieves a mean relative rank of 7.91%, a precision of 15.23%, a recall enhancement of 103.30, a hit-rate of 72.48%, a mean personality of 86.31%, and a mean novelty of 2.46. When used with threshold filtration network ($\delta=0.07$, $\gamma=0.9$), RWTF achieves a mean relative rank of 9.15%, a precision of 13.08%, a recall enhancement of 93.32, a hit-rate of 69.82%, a mean personality of 76.51%, and a mean novelty of 2.29. When comparing these criteria with those for the cosine similarity (Table 2), we clearly see their consistency. We therefore make the conjecture that the superior performance of the random walk model is not occasionally observed for some individual method for calculating user similarity. Moreover, we notice that the optimal performance achieved using cosine similarity (Table 2) is slightly better than that of Jaccard index (Table S1), suggesting that the cosine similarity is preferred by the random walk model and the network construction strategies for this dataset.

From Figures S2, S3 and S4, we observe that with Jaccard index, the network construction strategies and the related parameters influence the performance of the random walk model in similar patterns as those exhibited in Figures 4-6 (for cosine similarity measure), though the optimal values of the parameters may be different. For example, when used with power-law adjusted network, the mean relative rank decreases rapidly when *ß* increases from 1 to about 9, and then increases slowly when *ß* further increases. These observations again suggest that the high performance of the random walk model is consistent between different methods for calculating user similarities.

Consistency between different data sets

So far we have demonstrated the significant improvements of the proposed approach in making accurate and diverse recommendations using MovieLens. It is therefore natural to ask the question of whether such improvements are consistent between different data sets. To answer this question, we replace MovieLens with Netflix (5,000 users and 4,555 objects), repeat all the validation experiments with the use of cosine similarity measure, and summarize the results in Table S2. We clearly see from this table that the improvement in recommendation performance on Netflix data set is consistent with that exhibited on MovieLens (Table 2). For example, when used with power-law adjusted network ($\beta=6$, $\gamma=0.9$), RWPL achieves a mean relative rank of 6.07%, a precision of 8.59%, a recall enhancement of 61.76, a hit-rate of 49.94%, a mean personality of 90.34%, and a mean novelty of 2.48. When used with nearest neighbor network ($\lambda=0.05$, $\gamma=0.9$), RWNN achieves a mean relative rank of 6.19%, a precision of 9.13%, a recall enhancement of 64.60, a hit-rate of 51.32%, a mean personality of 89.64%, and a mean novelty of 2.34. When used with threshold filtration network ($\delta=0.11$, $\gamma=0.9$), RWTF achieves a mean relative rank of 6.74%, a precision of 7.54%, a recall enhancement of 56.01, a hit-rate of 47.01%, a mean personality of 87.62%, and a mean novelty of 2.45. These results suggest that the superior performance of the random walk model is not occasionally observed for some individual data set. Validation experiments with Jaccard index give us consistent results (Table S3).

Furthermore, we observe from Figures S5, S6 and S7 that on Netflix, the network construction strategies and related parameters influence the performance of the random walk model in similar patterns as those exhibited in Figures 4-6 (MovieLens), though the optimal values of the parameters may be different. Validation experiments with Jaccard index on Netflix give us consistent results (Figures S8, S9 and S10). These observations again suggest that the high performance of the random walk model is consistent between different data sets.

Finally, the above results are obtained based on relative small data sets (5,000 users). It is therefore natural to ask the question of whether the above observations are still valid for relatively large data sets. To answer this question, we increase the number of both sampled users and objects to 10,000, and we repeat the validation experiments. Not surprisingly, we observe similar patterns for accuracy, retrieval and diversity criteria on the large data sets (Tables S4, S5), suggesting that the previous conclusions are independent of the number of users and objects sampled.
